# Mixed-Linkage Glucan Is the Main Carbohydrate Source and Starch Is an Alternative Source during Brachypodium Grain Germination

**DOI:** 10.3390/ijms24076821

**Published:** 2023-04-06

**Authors:** Mathilde Francin-Allami, Axelle Bouder, Audrey Geairon, Camille Alvarado, Lucie Le-Bot, Sylviane Daniel, Mingqin Shao, Debbie Laudencia-Chingcuanco, John P. Vogel, Fabienne Guillon, Estelle Bonnin, Luc Saulnier, Richard Sibout

**Affiliations:** 1 INRAE, UR BIA, F-44316 Nantes, France; 2 DOE Joint Genome Institute, Berkeley, CA 94720, USA; 3 USDA-Agricultural Research Service, Western Regional Research Center, Albany, CA 94710, USA

**Keywords:** Brachypodium, grain, germination, cell wall, mixed-linkage glucan (MLG), lichenase

## Abstract

Seeds of the model grass *Brachypodium distachyon* are unusual because they contain very little starch and high levels of mixed-linkage glucan (MLG) accumulated in thick cell walls. It was suggested that MLG might supplement starch as a storage carbohydrate and may be mobilised during germination. In this work, we observed massive degradation of MLG during germination in both endosperm and nucellar epidermis. The enzymes responsible for the MLG degradation were identified in germinated grains and characterized using heterologous expression. By using mutants targeting MLG biosynthesis genes, we showed that the expression level of genes coding for MLG and starch-degrading enzymes was modified in the germinated grains of knocked-out *cslf6* mutants depleted in MLG but with higher starch content. Our results suggest a substrate-dependent regulation of the storage sugars during germination. These overall results demonstrated the function of MLG as the main carbohydrate source during germination of Brachypodium grain. More astonishingly, *cslf6* Brachypodium mutants are able to adapt their metabolism to the lack of MLG by modifying the energy source for germination and the expression of genes dedicated for its use.

## 1. Introduction

Grain germination is initiated by water uptake. The seed storage reserves are then mobilized by the action of hydrolytic enzymes to support germination processes and early seedling development. The hydrolytic enzymes are synthesized in the aleurone layer and in the scutellum, and secreted into the endosperm of germinating grains [1]. In most cereal grains examined to date, starch is the main storage compound synthesized in the endosperm and serves as the primary source of carbohydrate during germination and seedling growth, with the α-amylase enzyme playing a primary role in native starch granule degradation.

Grains of cereals such as wheat, maize, or sorghum, and most wild grasses have high starch contents (30–70%) and relatively low mixed-linkage (1,3;1,4) β-glucan (MLG) contents (generally <2%). Barley and oat have the highest MLG content among cereal grains (4–10%, *w*/*w*) and the lowest starch content [2]. In the same way, the grain of the wild grass *Brachypodium distachyon* (Brachypodium) from the reference accession Bd21, commonly used as a grass plant model, has been shown to have a particularly high MLG content (45%, *w*/*w*) and a very low starch content (6%, *w*/*w*). In addition, Brachypodium endosperm cell walls are much thicker when compared to other grass species [3,4]. These findings suggest that as well as having a structural role, MLG may supplement starch as a storage carbohydrate and may be mobilised during germination [3,5,6]. For unknown reasons, it seems that Brachypodium storage metabolism has shifted from starch to MLG compared with that of closely related grasses [7]. Genes encoding lichenases, degrading-enzymes specific to MLG, must exist to meet the nutrient requirement of seed germination, but so far, the lichenases responsible for the hydrolysis of MLG in the endosperm during germination have not been characterized in Brachypodium. In endosperm of germinated grain and coleoptiles of Brachypodium, Fan et al., 2022, highlighted the high expression of a lichenase they named BdLCH1 [8]. They also observed the strong expression of three lichenases specifically expressed in germinated endosperm, which could participate in the degradation of MLG during germination, but did not characterize them. The most described lichenases in grasses are the isoenzymes EI and EII from barley. Whereas EII was shown to be expressed exclusively in the germinated grains, EI was detected in a wider range of tissues during seedling development, such as endosperm, root, young leaves, and the scutellum at the beginning of the germination [9,10,11,12,13].

Highly prevalent in the Poales, MLG consists of an unbranched and unsubstituted homopolymer where β-1,4-linked glucose oligomers are connected by β-1,3 linkages in a nonrepeating but non-random fashion. The length and relative proportion of the oligomers vary among species but usually cellotrioses (DP3) and cellotetraoses (DP4) are dominant. The ratio DP3/DP4 determines the structure of MLG, and affects the solubility and visco-elasticity properties of the polymer [14]. Three cellulose synthase-like families, CSLF, CSLJ, and CSLH, have been implicated in the biosynthesis of MLG [15,16]. *CSLF6* is the most highly expressed *CSLF* gene in developing barley and wheat grain and the only one in Brachypodium [17,18]. BdCSLF6 is able to produce both of the linkages present in the (1,3;1,4)-β-D-glucan chain of MLG when expressed in *Pichia pastoris* or tobacco leaves [19]. CSLJ is exclusively found in *Hordeum vulgare* and *Triticum aestivum* and does not exist in Brachypodium [20]. Interestingly, higher transcript levels of CSLH were detected in vegetative organs of Brachypodium compared to cultivated cereals, suggesting that this synthase has a particularly prominent role in synthesis of MLG with a high DP3 to DP4 ratio [18].

To address the function of MLG in grasses, overexpression of *CSLF6* in barley, Arabidopsis, and Brachypodium were conducted, and the resulting plants had increased MLG content and exhibited detrimental effects on growth and development [21,22]. In the same vein, several mutants with reduced MLG content have been characterized, and confirmed that CSLF6 is the major isoform contributing to total MLG in both vegetative and floral tissues in barley [23,24,25,26,27], wheat [28], rice [29], and Brachypodium [30,31]. Although reductions in MLG content resulted in moderate reductions in vegetative growth and normal germination rates in rice, wheat, and barley, *cslf6* Brachypodium mutants presented a severe impact on survival, growth, and development [31]. Because homozygous mutant was not viable, the authors investigated heterozygous lines which showed 21% (*w*/*w*) lower grain MLG content. In what appears to be a compensatory mechanism, starch content was two times higher than in wildtype (WT) [31].

Due to the high MLG content in Brachypodium walls, it has been widely used to characterize this polysaccharide and explore its synthesis in grains and vegetative organs [3,5,32,33]. However, no data are available to date about MLG and cell wall changes occurring in grains during the germination process. To better understand its function as a storage polysaccharide, we explored the fate of MLG in germinating Brachypodium seeds. Using biochemical and immunological approaches, we showed that degradation of MLG begins a few days after germination. Lichenase activity was detected early in the germination process, and was maximal as soon as 48 h after imbibition. We identified three closely related hydrolases whose corresponding genes were highly expressed in the germinated grain, and characterized lichenase activity for two of them. By using new Brachypodium mutants [34], we demonstrated that while plants mutated in the *CSLF6* gene were deficient in MLG, they were viable with a germination rate similar to the wild type and an only slightly slower seedling growth. The grains from mutant plants had thinner cell walls and higher contents of starch in the endosperm cells and outer layers. This phenotype is accompanied with a decrease of lichenase expression level and an increase of amylase gene expression, indicating that mutant plants compensated for the new source of storage carbohydrate. Inversely, no clear phenotype was detected for the grains affected in the *CSLH1* gene.

## 2. Results

### 2.1. Histology and Polysaccharide Composition of Wild Type Brachypodium Grain during the Germination

**Figure 1:**
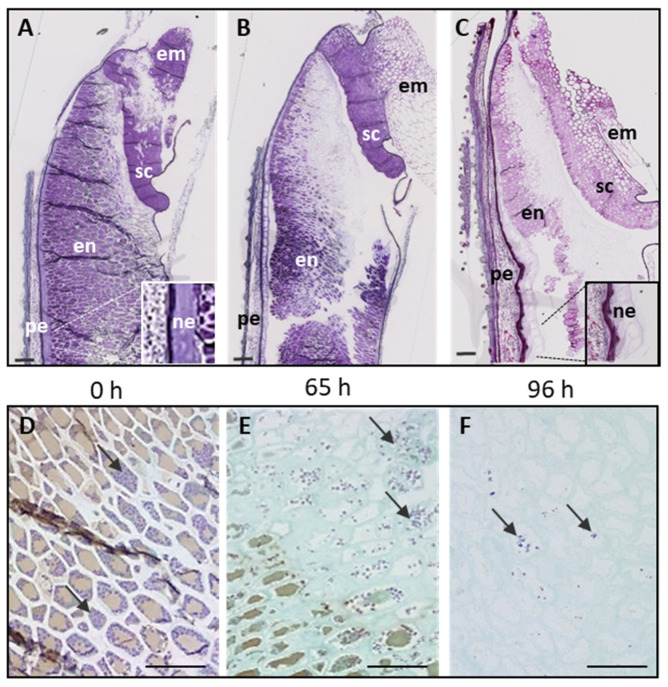
Histology of Brachypodium grains at 0, 65, and 96 h after the beginning of germination. Images (**A**–**C**): longitudinal cross-sections of grains stained with Toluidine blue. Magnifications indicate the nucellus epidermis. Images (**D**–**F**): endosperm cells stained with iodine and Toluidine blue. Starch granules are coloured purple by iodine while the other cellular components appear turquoise and brown with Toluidine blue. Arrows indicate starch granules. Scale bar = 100 µm. en: endosperm; pe: pericarp; ne: nucellar epidermis; em: embryo; sc: scutellum.

After four days of imbibition in cold water, longitudinal WT grain sections were examined at different times between 0 h and 96 h after the start of germination. Note that the latter stage in our time course includes the beginning of the seedling stage since root and radicle had emerged from the grains. Toluidine blue staining revealed changes in the cellular content according to the stage of germination and the location in the grain (Figure 1A–C). Indeed, we detected a decreasing gradient of Toluidine blue staining intensity throughout germination, suggesting a progressive degradation process that began in the endosperm near the germ and continued toward the peripheral region. Iodine staining revealed relatively few starch granules that progressively disappeared during germination (Figure 1D–F). No changes were observed in the epicarp and mesocarp cells. In contrast, the nucellar epidermis appeared progressively less stained with Toluidine blue, suggesting a strong degradation of its cell wall compounds in the course of germination (magnifications Figure 1A,C).

**Figure 2:**
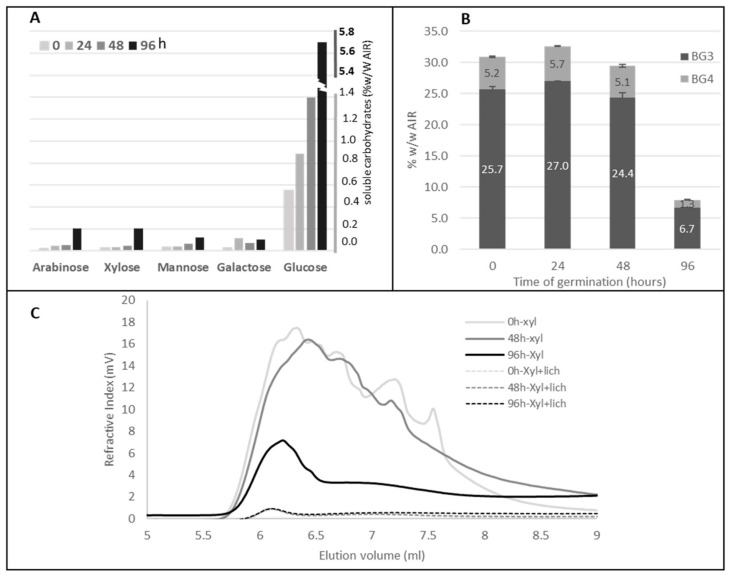
Biochemical analysis of germinated grains (**A**). Composition of soluble sugars obtained from Brachypodium wild type grains during the germination process (hours counted after the imbibition step). (**B**). HPAEC analysis of tri- and tetramers (BG3 and BG4, respectively) released from MLG after lichenase treatment of the AIR of germinated WT grains. (**C**). HPSEC analysis of hemicelluloses extracted by alkaline treatment from grain at various germination times and after xylanase digestion to detect the MLG profile, or xylanase + lichenase digestion to degrade both arabinoxylans and MLG.

In order to quantify polysaccharide modification during germination, the sugar composition of both alcohol-soluble and alcohol-insoluble residue (AIR) of imbibed and germinated grains was determined (Table 1; Figure 2A). The total sugar content decreased during germination (Table 1). This decrease is mainly due to the release of glucose which is the main sugar found in the polysaccharides of Brachypodium grains [3,5,33]. The starch content between 48 and 96 h of germination decreased as observed by histological staining (Table 1; Figure 1D–F). High-performance anion-exchange chromatography (HPAEC) analysis of 3-O-β-cellobiosyl-D-glucose (BG3) and 3-O-β-cellotriosyl-D-glucose (BG4) tri- and tetrasaccharides released by lichenase from grains at the different stages of germination revealed a sharp decrease of MLG content, especially after 96 h (Table 1; Figure 2B), which was confirmed by HPSEC analysis (Figure 2C). Interestingly, the molecular weight of MLG in grain at 48 h after the start of germination was found to be lower than that of the imbibed grain (Figure 2C). This suggests an onset MLG degradation between 0 and 48 h of germination, although not yet detected by the analysis of MLG content in the AIR (Table 1). Although highly degraded, a non-negligible amount of MLG was still present 4 days after the start of germination, which could correspond to residual endosperm MLG or other MLG present in the pericarp of the grain.

**Figure 3:**
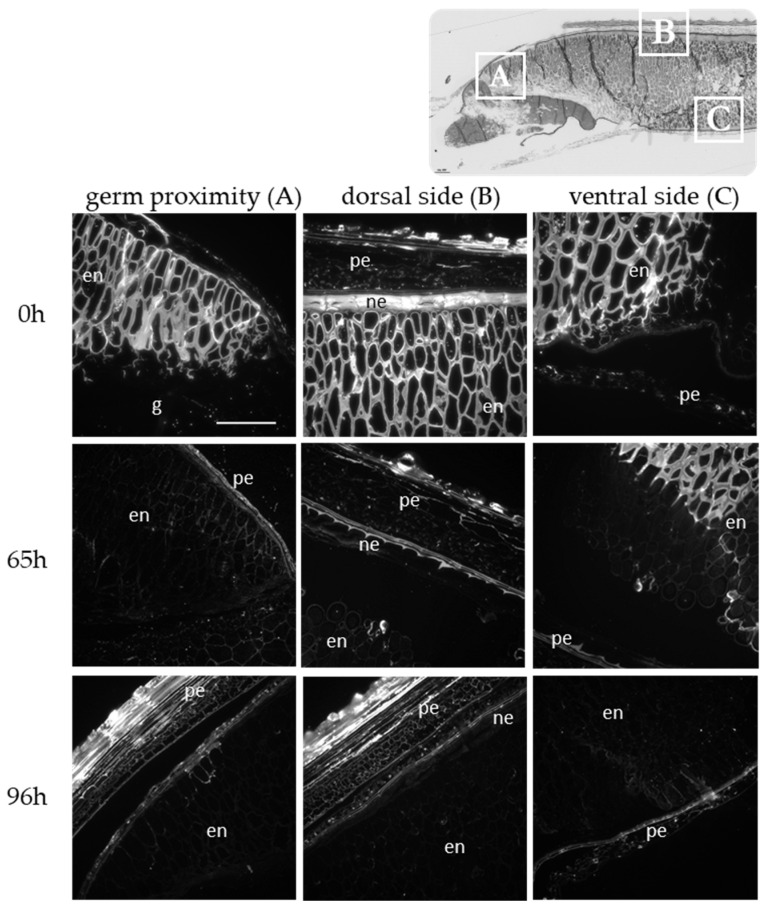
Immunolabeling of germinated grains (0, 65, and 96 h after imbibition) using antibodies specific to MLG. Scale bar = 50 µm. g: germ; pe: pericarp; en: endosperm; ne: nucellar epidermis.

To verify this hypothesis, we monitored the degradation of MLG during germination at the tissue level. Immunolabellings were performed on sections of germinated grain using specific antibodies (Figure 3). A negative control with lichenase pretreatment is provided for each stage in Supplementary Figure S1. A strong fluorescence was detected in the cell walls of endosperm and in the pericarp in all area of the imbibed grain, as previously shown for non-germinating grain [33]. Forty-eight hours after the start of germination, only cell walls of the endosperm distant from the germ and at the ventral side of the grain were still highly labelled. The endosperm close to the germ and in the middle of the grain as well as the nucellar epidermis were clearly less fluorescent, reflecting a loss of recognition of MLG epitopes by the antibodies. After 96 h of germination, no more fluorescent signals were detected in the endosperm or in the nucellus epidermis, but a signal persisted in the pericarp. The degradation of MLG in both the endosperm and nucellar epidermis confirms their role as storage polysaccharides for germination and early seedling growth.

Contrary to glucose, the proportion of galactose, xylose, and arabinose was found to increase during germination, probably due to the decrease in glucose (Table 1). Arabinose and xylose are found mainly in arabinoxylans in Brachypodium grains [33]. Beside this increase, a slight release of xylose and arabinose was observed in the soluble material from germinated grain (Figure 2A), most likely reflecting a weak and late degradation of arabinoxylans during germination.

### 2.2. Lichenase Activity in Germinated Grains 

**Figure 4:**
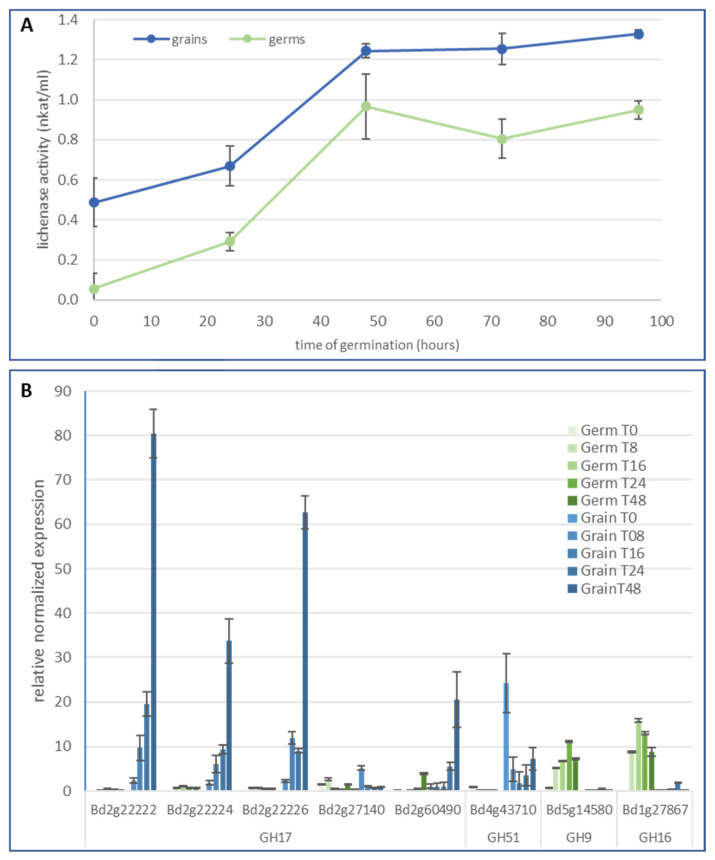
(**A**). Lichenase activity measured from protein extracts of germinated grains (without germ) and germs. Lichenase activity was determined by the Nelson method and barley MLG was used as substrate. (**B**). Transcript levels (qRT-PCR) of Brachypodium genes encoding putative lichenases during the germination (0, 8, 16, 24, and 48 h of germination) in grains and germs. Error bars represent standard deviation of the mean (*n* = 3).

Since significant degradation of MLG was observed in the cell walls of the endosperm and nucellar epidermis in germinated grains, we monitored and characterized the corresponding enzymatic activity. MLG from barley was used as a substrate to follow lichenase activities during germination in both grain and germ (Figure 4A). After the imbibed step, a significant lichenase activity was detected in the grain while it was almost non-existent in the germ. Lichenase activity increased progressively until 48 h after the start of germination and was relatively stable between 48 and 96 h of germination. It was higher in the grain than in the germ but followed a similar time course along germination.

According to the CAZY database, the glycoside hydrolase families in which lichenase activities have been referenced are GH5, GH9, GH16, GH17, GH26, and GH51. Among these GH families, we selected gene candidates with high expression levels in germinated grain (http://bar.utoronto.ca/efp_brachypodium/cgi-bin/efpWeb.cgi (accessed on 15 Februar 2023); [35]) or those already known to encode proteins with bona fide lichenase activity (Supplementary Figure S2). Five genes were selected in the *GH17* family, four of which are particularly well expressed in germinated grains according to the RNAseq data, and the fifth one is already characterized as lichenase (Bradi2g27140 named *BdLCH1*; [8]). Separated germs and grains were selectively analyzed by quantitative RT-PCR (qRT-PCR). Transcript levels of Bradi2g22222, Bradi2g22224, and Bradi2g22226 substantially increased during germination in the grain (Figure 4B). By contrast, these genes were very weakly expressed in the corresponding germs. These results are in line with those of Fan et al. (2022) [8] except for *BdLCH1* that we found poorly expressed during germination. Our result is, however, consistent with those coming from eFP browser (Supplementary Figure S2). At last, a relative high expression was also observed for the Bradi2g60490 gene in the grain. In the germ, all tested *GH17* genes were poorly expressed. This is also the case for the gene Bradi2g43710 (*GH51* family), for which the expression was, however, relatively high at the imbibition step in grain, but remained weak and steady during germination. Genes from both the *GH9* and *GH16* families (Bradi5g14580 and Bradi1g27897, respectively) were found expressed in the germ. In the grain, transcripts were undetectable or found at very low levels.

**Figure 5:**
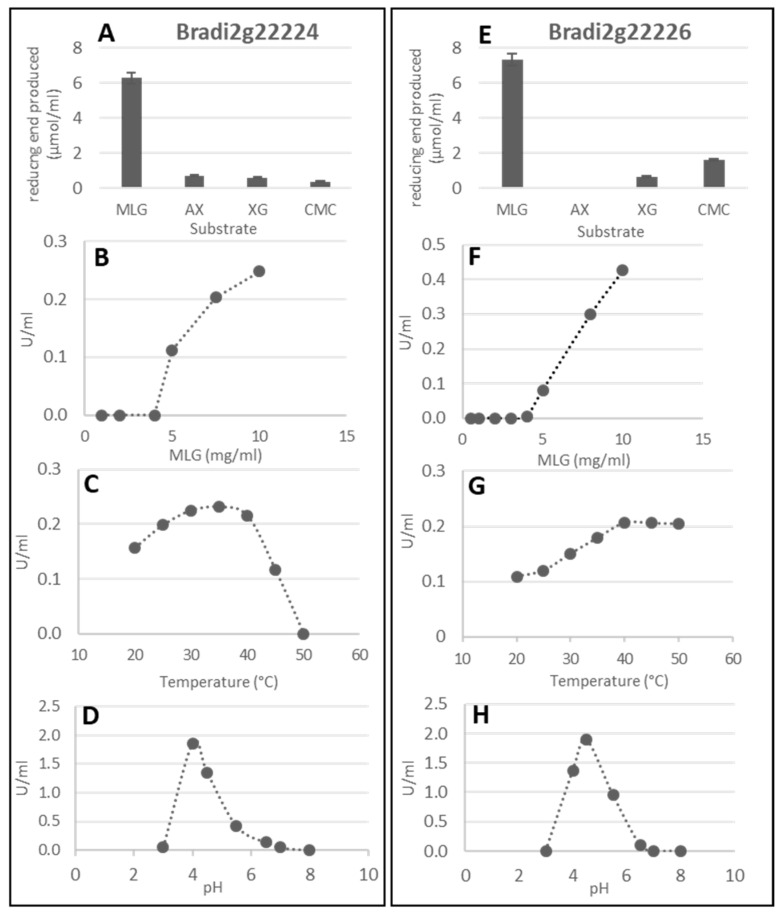
Enzymatic activity of the recombinant enzymes encoded by Bradi2g22224 and Bradi2g22226. Enzymes were incubated with 10 mg/mL of substrate during one night for substrate specificity determination (**A**,**E**) or 2 h for other kinetics based on substrate concentration (**B**,**F**), temperature (**C**,**G**), and pH (**D**,**H**). MLG: barley mixed linkage glucan; AX: wheat arabinoxylans; XG: tamarin xyloglucan; CMC: carboxymethylcellulose.

Among the *GH17* genes, the *BdLCH1* sequence is rather similar to that of the well described barley EI lichenase, while Bradi2g22224 and Bradi2g22226 have more sequence similarities with the EII lichenase (Supplementary Figure S3A). Interestingly, Bradi2g22222 and Bradi2g60490 are genetically most distant from the two barley lichenases. Thus, we characterized the enzymatic activity of the corresponding recombinant protein encoded by Bradi2g22222, Bradi2g22224, and Bradi2g22226. These have a very high sequence identity but Bradi2g22222 is the more distant with slight amino acid changes (Supplementary Figure S3B) and a predicted higher isoelectric point (pI 9.13 vs. pI 6.24 and pI 5.2 for Bradi2g22224 and 2g22226, respectively). To establish their lichenase activity, the proteins were expressed using a *Pichia pastoris* expression system (Supplementary Figure S3C). The substrate specificity of the enzymes was verified by using other polysaccharidic substrates (arabinoxylans from wheat, xyloglucan from tamarin, and carboxymethyl cellulose) (Figure 5A,E and supplementary Figure S3D for Bradi2g22222). For both Bradi2g22224 and Bradi2g22226 enzymes, the optimal pH of the lichenase activity was determined around 4-4,5 (Figure 5D,H), which is little lower than the values reported for the recombinant BdCLH1 expressed in *E.coli* [8] and lichenases extracted from barley (EI and EII, [9]), for which the optimal pH was 5. The optimal temperature was found to be 35 °C for Bradi2g2224 and 40 °C for Bradi2g22226 (Figure 5C,G). Interestingly, the latter still showed activity until 50 °C. The results demonstrated a specific lichenase activity of the Bradi2g2224 and Bradi2g2226 recombinant proteins when expressed in *Pichia pastoris*, highly suggesting their involvement in the degradation of MLG during germination. Enzymatic activity of the purified proteins was performed by the measuring of the amount of reducing ends produced from barley MLG as substrate. In contrast to Bradi2g22224 and Bradi2g22226, the recombinant protein Bradi2g22222 degraded very little MLG in any of the assay conditions tested (various temperatures, pH, and buffers).

### 2.3. Characterization of Brachypodium Mutants Affected in Genes Involved in MLG Biosynthesis

In order to investigate the role of MLG during germination, we decided to study Brachypodium plants with decreased or no MLG in their cell walls. We therefore selected mutants in two genes encoding synthase-like cellulose, *CSLF6* and *CSLH1*, potentially involved in the MLG biosynthesis [17,18], from a collection of sequenced Brachypodium mutants [36]. Two mutant lines were chosen, NaN1812 and NaN212, for which mutations are located at splice sites, with strong predicted impacts on CSLF6 and CSLH1 function (Supplementary Figure S4A).

**Figure 6:**
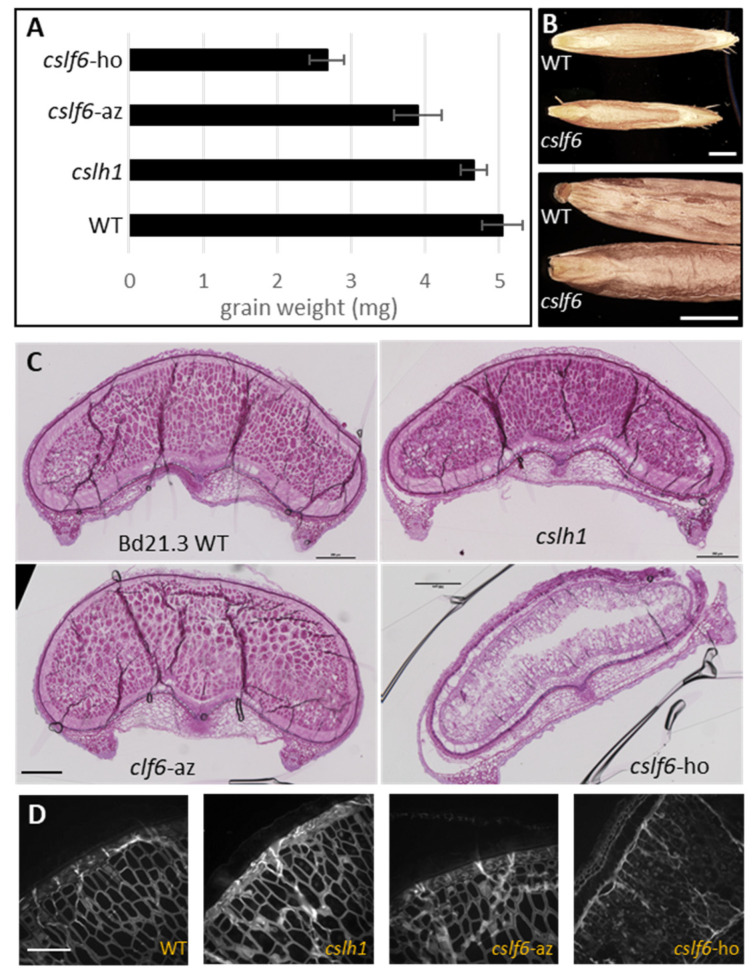
Morphological characterization of mutant grains. (**A**): Average weights of wild type and mutant grains (mean of 10 grains, *n* = 10). (**B**): Stereomicroscopic images of wild type and homozygous *cslf6* mutants showing the size and external aspect of grain. Scale bar = 1 mm. (**C**): Cross-sections of wild type and mutant grains (15 days of flowering) stained with Toluidine blue. Scale bar = 200 µm. (**D**): Calcofluor staining of wild type and mutant grains (15 days after flowering). Scale bar = 50 µm. *cslf6*-ho: homozygous *cslf6* mutant; *cslf6*-az: azygous *cslf6* mutant.

Both *cslf6* and *cslh1* homozygous mutants were viable and able to grow and form mature grains. No apparent phenotype was detected for the homozygous *cslh1* mutant plants. In contrast, homozygous *cslf6* mutants were dwarf-sized with white patches in the leaves while the corresponding azygous (plant segregated from the same mutagenized line with a wild type allele for *CSLF6*) did not (Supplementary Figure S4B). We also noticed a reduced weight and wrinkled appearance of the *cslf6* grains (Figure 6). A similar phenotype was previously described for another *cslf6* mutant [31]. Grain sections stained with Toluidine blue or calcofluor evidenced a much lower staining of the cell walls of the homozygous *cslf6* mutant, reflecting a thinner cell wall (Figure 6).

#### 2.3.1. The *cslf6* Mutant Grains Are Completely Devoid of MLG

**Figure 7:**
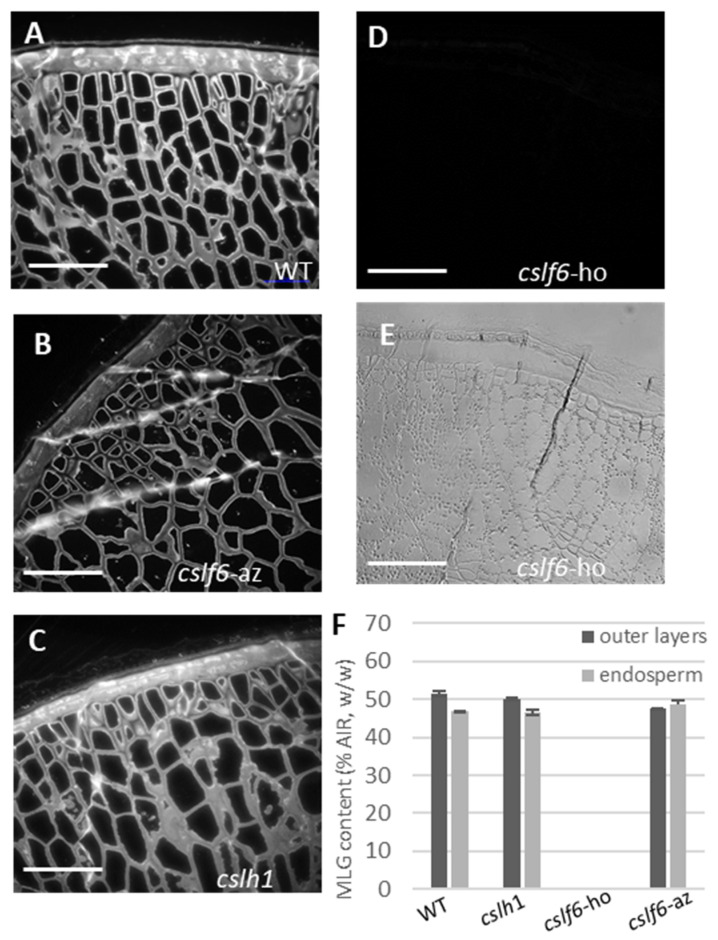
(**A**–**D**): Fluorescence immunolabeling of MLG in a grain cross-section of WT, *cslh1*, azygous (az), and homozygous (ho) *cslf6* mutants (15 days after flowering). The homozygous *cslf6* (*cslf6*-ho) does not show any fluorescence signal (**D**). (**E**): Differential interference contrast of the *cslf6*-ho mutant. Scale bar = 50 µm. (**F**): MLG content in the endosperm and pericarp of WT and mutant grains (15 days after flowering) expressed as a percentage of AIR (*w*/*w*).

To assess the impact of the *cslf6* and cslh1 mutations on MLG biosynthesis, the MLG content from the AIR samples of the developing grains was determined and immunolabelling was performed on the grains (Figure 7). Grains from the wild type, azygous *cslf6* and homozygous *cslh1* mutants contained a similar amount of MLG in the cell wall of outer layers and endosperm, which was estimated to be around 50% of the AIR; however, we noticed the BG3/BG4 ratio was slightly lower in the endosperm of the *cslh1* mutant (Table 2). In contrast, the grains of the homozygous *cslf6* mutant were completely devoid of MLG in their cell walls (Figure 7F). Immunolabelling performed on grain sections with a specific MLG antibody confirmed the absence of MLG in the grains of the *cslf6* mutant both in outer layers and the endosperm (Figure 7D). Cellulose and arabinoxylans were detected in the grain of *cslf6* mutant, although less intensively due to the thinness of the cell walls (Supplementary Figure S5). Analysis of the sugar composition revealed a higher percentage of arabinose and xylose content in the endosperm and in the pericarp of the developing grain as well as in the dried grains of the *cslf6* mutant (Table 2). Assuming that arabinose and xylose are mainly derived from arabinoxylans, these would constitute the majority of cell wall polysaccharides in the *cslf6* mutant.

#### 2.3.2. Loss of MLG Comes with an Increase of Starch in the *cslf6* Mutant

**Figure 8:**
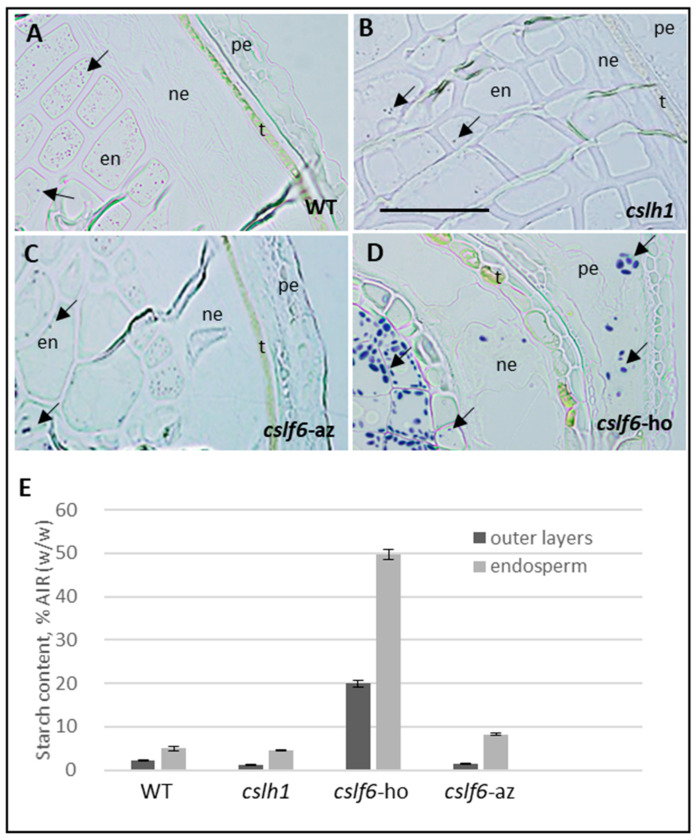
Localization of starch in grains of *cslf6* and *cslh1* mutants and controls (WT and azygous *cslf6*). (**A**–**D**): Bright-field images of grain cross-sections stained with iodine. Scale bar: 50 µm. pe: pericarp; ne: nucellar epidermis; en: endosperm; t: testa. (**E**): Total amount of starch expressed as a percentage of AIR (*w*/*w*). Arrows indicate starch granules. *cslf6*-ho: homozygous *cslf6* mutant; *cslf6*-az: azygous *cslf6* mutant.

The starch content in mature grains of mutants was assayed by high-performance anion-exchange chromatography (HPAEC). Starch content, which accounts for 5% of the AIR (*w*/*w*) in WT and azygous *cslf6* grains, is five-fold more abundant in *cslf6* (Table 2). In the endosperm of the wild type and of azygous *cslf6*, it represented 5 to 8% of AIR (*w*/*w*) and up to 50% in the endosperm of the homozygous *cslf6* mutant. Starch was present in low amounts in outer layers of the wild type, azygous *cslf6*, and homozygous *cslh1* mutants (1.5–2% AIR *w*/*w*) but accounted for up to 20% of the AIR in the homozygous *cslf6* mutant (Table 2 and Figure 8E). While the low amount of starch in the wild type could be attributed to a contamination during manual separation of endosperm and outer layers, it is unlikely that the amount of starch in the outer layers of the *cslf6* mutant is solely due to contamination. To check the possibility of starch accumulation in outer layers of the *cslf6* mutant, we performed iodine staining on developing grain sections (Figure 8A–D). As expected, small starch granules were found located only in the endosperm of wild type, *cslh1*, and azygous *cslf6* (Figure 8A–C; [37]). In contrast, the homozygous *cslf6* mutant contained not only a huge amount of large starch granules in the endosperm but also in the epidermal cells of the nucellus and in the pericarp (Figure 8D).

#### 2.3.3. *cslf6* Mutation Impact on Germination and Seedling Growth

**Figure 9:**
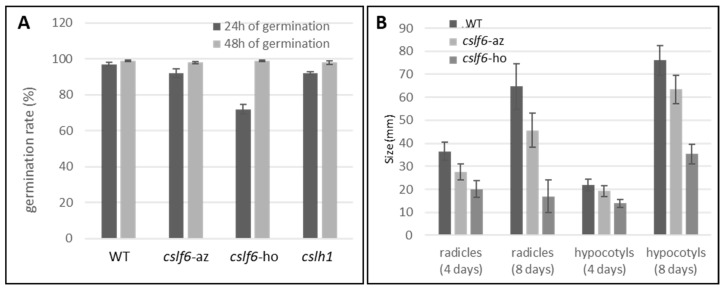
Development of germinated lines. (**A**). Germination rate at 24 and 48 h of germination of *cslf6* and *cslh1* mutants and controls (WT and azygous *cslf6*). (**B**). Size of radicles and hypocotyls of a *cslf6* mutant compared to controls (WT and azygous *cslf6*). n = 100 grains. *cslf6*-ho: homozygous *cslf6* mutant; *cslf6*-az: azygous *cslf6* mutant.

In order to assess the impact of MLG loss on grain germination process, the germination rate of the different plant lines was studied (Figure 9). Forty-eight hours after the beginning of germination, all the examined lines of Brachypodium showed almost 100% germination. However, at 24 h, a significantly lower germination rate was observed for the homozygous *cslf6* (Figure 9). These results indicate that the grains of the *cslf6* mutant have a similar germination capacity to the wild type but their germination is slower. Additionally, radicle and hypocotyl sizes of four- and eight-days-old homozygous *cslf6* mutant seedlings were significatively smaller, indicating a slower growth rate in young *cslf6* mutant seedlings (Figure 9). Eight days after the beginning of germination, the plants were transferred to a phytagel medium with or without their grain parts (endosperm and pericarp) and monitored for few days (Supplementary Figure S6A,B). In the presence of the whole grain, the hypocotyl size of homozygous *cslf6* mutant plants was clearly reduced compared to the corresponding azygous and wild type, indicating that the mutation affects both germination, seedling, and plant growth efficiency. In contrast, no difference in hypocotyl growth was observed when the germs were removed from the grain (Supplementary Figure S6B). Without any storage reserves contained in the endosperm and outer layers, plants stopped growing even for the wild type and the azygous *cslf6*.

**Figure 10:**
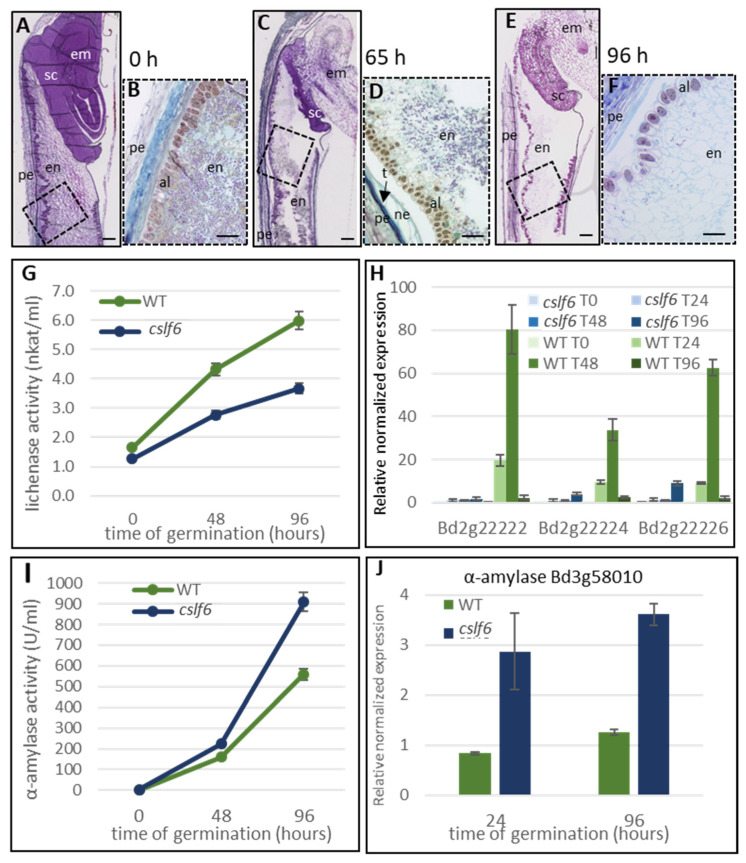
Catabolism of reserve sugars in homozygous *cslf6* mutant. (**A**–**F**): Histology of *cslf6* grains at 0 h (images (**A**,**B**)), 65 h (images (**C**,**D**)), and 96 h (images (**E**,**F**)) of germination. (**A**,**C**,**E**): Images of longitudinal grain cross-sections stained with toluidine blue. (**B**,**D**,**F**): Higher magnification images of the sections corresponding to the dotted boxes on the images (**A**,**C**,**E**), respectively, and stained with both Toluidine blue and iodine. Starch granules are coloured purple by iodine. Scale bar = 100 µm. en: endosperm; al: aleurone; pe: pericarp; t: testa; ne: nucellar epidermis; em: embryo; sc: scutellum. (**G**): Lichenase activity from wild type and *cslf6* germinated seeds. (**H**): Transcript levels of Bradi2g22222, Bradi2g22224, and Bradi2g22226 genes in WT and *cslf6* grains at 0, 24, 48, and 96 h of germination. (**I**): α-amylase activity in WT and *cslf6* mutant grains at 0, 48, and 96 h of germination. (**J**): α-amylase transcript level in WT and *cslf6* mutant grains at 24 and 96 h of germination. Error bars represent standard deviation of the mean (*n* = 3).

To check if starch or other non-starch polysaccharides are able to counterbalance the storage function of MLG in the *cslf6* mutants, changes in the grain sugar composition during germination were followed (Table 3). Except for glucose, the percentage of neutral sugars remained stable or even increased such as xylose, arabinose, and galactose, as previously shown for wild type germinated grains (Table 1). This suggests that the other cell wall polysaccharides such as arabinoxylans would not serve as source of energy for germination of *cslf6* mutant grains. Ninety-six hours after the start of germination, a significant decrease in starch content took place (Table 3). The degradation of starch during germination of *cslf6* grain was confirmed by histological staining (Figure 10A), which strongly suggests that starch takes the place of MLG, at least partially, as a storage reserve in the *cslf6* mutant.

Since a switch between MLG and starch occurs in the *cslf6* mutant grains, we monitored the enzyme activities responsible for the degradation of these two polysaccharides throughout germination. Using barley MLG as substrate, we showed that lichenase activity was clearly lower during germination of homozygous *cslf6* mutant grains compared to the wild type (Figure 10B). The expression level of the genes we previously identified as being primarily responsible for lichenase activity during germination was found to be particularly low in the *cslf6* mutant (Figure 10C). Inversely, a significantly higher activity of starch degradation during the germination of the *cslf6* mutant was detected (Figure 10D). On the basis of sequence homology and transcriptomic data (http://bar.utoronto.ca/efp_brachypodium/cgi-bin/efpWeb.cgi (accessed on 15 Februar 2023); [35]), we identified an α-amylase gene belonging to the *GH13* family, Bradi3g58010, highly and specifically expressed in germinated grain (Supplementary Figure S2) and thus strongly predicted to be involved in starch degradation during germination. The expression level of this α-amylase gene was seen to be three times higher in the germinated grains of the *cslf6* mutant compared to the wild type 24 and 96 h after the start of germination (Figure 10E), which corroborates the increase of starch in the *cslf6* mutant. It should be noted that the expression level of lichenase-encoding genes was sharply decreased after 96 h of germination in the wild type (Figure 10C), while lichenase activity still increased (Figure 10B). This could be explained by the temporal gap between transcription and translation events. In the same way, we noticed that α-amylase activity in wild type and *cslf6* at 48 h of germination was rather similar (Figure 10D), while gene expression at 24 h strongly increased in *cslf6* compared to wild type (Figure 10E).

## 3. Discussion

The wild grass *Brachypodium distachyon* is an important plant model that has led to fundamental advances in grass biology [38]. Detailed studies of grain development has revealed similarities with other grasses but also some interesting features [3,5,6,33,39]. The unusual thickness of endosperm and nucellar epidermis cell walls particularly abundant in MLG and a low starch content are the main ones. Until recently, the question of why such a high amount of MLG is present in the Brachypodium grain cell walls remained unclear. Several authors have suggested that MLG serves as a source of nutrient for seed germination and seedling establishment [3,5,6]. However, to our knowledge, this hypothesis was never tested. In the present work, we analyzed Brachypodium grain throughout germination and seedling growth and showed that MLG was massively degraded to provide the energy necessary for growth. We identified some of the enzymes in the metabolic pathway. Noticeably, a clear-cut transition from non-degraded to degraded endosperm in a gradient from the proximal to the distal end of the grain was observed during germination. This mechanism has also been reported in other grasses, like barley, where this transition was formerly described as an enzymatic front that migrated centripetally from scutellum and aleurone inwards [40]. Our biochemical analyses confirmed that MLG is the main component of the cell wall to be degraded and it is reasonable to attribute the thinning of the endosperm cell wall to this mechanism. Again, thinning of cell walls throughout germination has been observed in many plants, such as in barley grains and coffee beans [41,42]. Opanowicz et al. estimated that the cell wall thickness in the central endosperm of Brachypodium grain was about 4.4 µm, which is more than twice the thickness of the cell walls in the central endosperm of wheat grain [6]. They showed that cell walls thinned to 2.4 μm during germination and that they were depleted of calcofluor reactive material, which corroborates our results. The nucellar epidermis, unusually prominent in Brachypodium, was also modified during germination. It may act as a reserve storage tissue as previously suggested [6,43]. It would be interesting to analyze separately the endosperm and the outer layers of germinated grains to determine precisely when MLG is degraded during germination in these tissues. It is highly probable that endosperm MLG is digested first to supply nutrients for early germination, and then MLG in nucellar epidermis can be degraded later to meet later needs during seedling growth (our work; [43]).

Four days after germination, little MLG remains in the grain cell walls. This could be residual MLG from the endosperm, but our immunostaining data suggest that it is more likely MLG in the epicarp and mesocarp cell walls. The persistence of MLG in the outer layers of germinated grains is consistent with a structural and protective function of MLG in these tissues [44]. We observed that *cslf6* grains, which contain no detectable MLG, have thinner cell walls, show a wrinkled appearance on their surface, and are more brittle. Previous proteomic analysis of the outer layers in wheat grains revealed the presence of enzymes whose functions are likely involved in tissue strength and subsequently in the protection of the embryo and the endosperm against pathogens but not in the supply of nutrients for germination [45]. However, in the case of Brachypodium, the nucellar epidermis could combine both storage and protective functions. A more detailed study targeting the nucellar epidermis throughout germination would lead to better understanding of the role of this particularly prominent cell layer in Brachypodium.

Cell wall degradation is common during germination but not necessarily for the purpose of providing a source of nutrients. Indeed, cell walls are usually associated with structural, hardness, or water-related functions [46]. During germination, breaking down this physical barrier is most likely required to allow access of hydrolytic enzymes to substrates within the cell, such as proteins or starch [47]. In germinating barley, MLG content decreases along with starch degradation, but the potential role of degraded MLG as a significant energy source for germination has not been considered; rather, MLG degradation is thought to simply be necessary for accessibility to protein and starch reserves [48]. In the case of Brachypodium, the particularly high abundance of MLG and the thickness of the endosperm cell wall strongly indicate that cell wall degradation is more likely used to produce nutrients for germination than solely to facilitate access to starch and proteins. While unusual in grasses, the use of cell wall polysaccharides as a major source of storage carbohydrate in grains and seeds is not confined to Brachypodium. Species of Bromus also possess thickened endosperm walls and a reduced starch content [7]. Moreover, a significant number of dicotyledonous seeds uses cell wall polysaccharides rather than starch as the main storage polymers in the endosperm. These include mannans in coffee, lettuce, and tomato, glucomannan in orchids, galactomannans in fenugreek seed, xyloglucans in tamarind, and nasturtium cotyledons [49,50,51]. The use of cell wall polysaccharides as storage carbohydrates required an evolutionary adaptation that impact their metabolism, such as temporal displacement between biosynthesis and hydrolysis, and/or increase in their proportion and structure [46].

Contrary to MLG, arabinoxylans were only slightly degraded four days after germination. These results align with those of previous studies on barley and would indicate a distinct function of this hemicellulose during germination [41,52]. In a recent study, a new cell wall model was proposed for barley endosperm where arabinoxylans and pectins make up the outer cell wall layer, whereas inner layers are made of MLG [41]. Using confocal laser scanning microscopy and specific fluorescent stains, authors demonstrated that germination results in the loss of MLG staining, suggesting that the MLG were strongly hydrolyzed, whereas arabinoxylan layers remained visually intact. The structure of arabinoxylans is more complex than those of MLG and their degradation is more complex than MLG.

The hydrolysis of MLG into sugars requires the activity of lichenases. A handful of lichenases from grasses have been characterized [10,11] (barley); [53] (rice); [54] (wheat); [55] (oat); [56] (maize); [8] (Brachypodium)). Lichenase homologs are often expressed within specific tissues and with temporal expression patterns. They most often belong to the GH17 family [57]. This is the case for two very similar homologs in barley, EI, specific to the germinated grain, and EII, expressed in a wide range of tissues [9,11,12,13]. A recent study showed the lichenase BdLCH1, highly abundant in the coleoptiles and germinated grain, might be related to the EI enzyme in barley [8]. We detected only a very low expression of the *BdCLH1* gene in germinated grain until 48 h after imbibition, which was consistent with the eFP transcriptomic data (Supplementary Figure S2). In contrast, we found a particularly high and specific expression of the homologs Bradi2g22222, Bradi2g2224 and Bradi2g22226 in germinated grains as previously reported by Fan et al. (2022) [8]. It is likely that the expression of these three homologs allows for increased lichenase activity during germination, unless each homolog is regulated in a tissue-specific and time-dependent manner. In the current work we demonstrated that at least two of them are able to hydrolyse barley MLG when expressed heterologously in *Pichia pastoris*. Surprisingly, we were not able to detect significant hydrolytic activity on MLG for Bradi2g22222 despite in silico lichenase activity prediction. Although we cannot rule out that the addition of the C-terminal His tag disrupted the activity of the enzyme. Curiously, several recombinant proteins presenting high sequence similarities with the maize lichenase MLGH1 also failed to degrade MLG in vitro despite their lichenase activity prediction [56]. Such predicted lichenases that fail to degrade MLG in vitro may suggest enzymatic activities under specific conditions.

Our experimental data do not show any significant correlation between MLG content and expression of *CSLH1* during endosperm maturation. This is consistent with the major contribution of the *CSLF6* gene to MLG content within the grain [58]. However, it seemed that the ratio BG3/BG4 was slightly lower in the endosperm of developing grain of the *cslh1* compare to those of wild type, which supports the hypothesis of a potential structural role for MLG produced by CSLH1. In barley grain, transcript profiles showed that *CSLH1* peaks at four days after pollination (DAP), one day before the appearance of MLG in endosperm cell walls and then declines to low levels by 8 DAP, implying its involvement in early endosperm development [15]. In contrast, data from eFP browser indicate more *CSLH1* transcripts, although at very low level, in mature and germinated grains of Brachypodium (Supplementary Figure S2). Further investigations at the enzymatic level and localization of gene expression are needed to confirm the potential involvement of CSLH1 in the DP ratio of MLG in the endosperm.

We showed that plants with a mutation in *CSLF6* were totally devoid of MLG but, astonishingly, they were viable and able to form grains that germinated with only slightly reduced vigor. We thus clearly showed that MLG is not essential for Brachypodium plant survival. This corroborates previous findings described for other grasses such as barley or rice [23,25,29]. In contrast, a recent study on Brachypodium indicated that the loss of CSLF6 capacity was detrimental to plant development with a negative impact on seed set, severely stunted growth and the absence of any viable seed [31]. In light of our results, the deleterious effect they observed could rather be due to presence of other mutations on their genome. Although we did not provide complementation data fully demonstrating that mutation in CSLF6 was the only cause of the MLG/starch balance phenotype that we observed, the fact that the loss of the major seed storage polysaccharide, MLG, had only a minor impact on plant growth highlights the resiliency of Brachypodium. In this case, seed survival and germination rates are rescued by the concomitant increase of starch content in *cslf6*. Furthermore, although the cell walls were thinner and starch content was higher in the grains, we did not observe an increase in endosperm cell size in the *cslf6*. Our results do not corroborate data from Trafford et al. that suggest that limited increase in cell size may be the consequence of cell-wall thickening [4]. In the light of our results, starch accumulation also does not appear to drive cell expansion.

Several studies of mutants with loss of function of *CSLF6* indicated an increase in starch content in response to the decrease of MLG in the endosperm ([23,25,29,31]; this work). Inversely, the overexpression of *CSLF6* induced a decrease in starch content in addition to the increase of MLG [21,22]. The primary cause of this metabolic shift is not known. It was suggested a possible balanced regulation or a competition between MLG and starch synthesis in the endosperm, both starch and MLG being synthesized from the common cytosolic UDP-glucose substrate [4,57]. It now appears crucial to pinpoint the regulatory factors of these two metabolic pathways to understand this cross-regulation. Even if starch increase in the endosperm of the *cslf6* mutant did not fully restore growth efficiency, it still allows germination and ensures viability of plants. It is likely that *cslf6* compensates for the loss of MLG by degrading starch molecules during the germination to provide energy for germination and seedling growth. At last, we reported a correlation between the transcript level of select carbohydrate degrading enzymes and their substrates during germination. Indeed, we observed an increase of α-amylase and a decrease of lichenase transcripts in the germinated grain in response to the shift between starch and MLG in *cslf6*. This likely results from a carbon-source dependent regulation of these catabolic enzymes during germination. The mechanisms of this regulation remain to be elucidated.

Starch granules were also observed in the *cslf6* pericarp tissues while none was observed in the wild type grain at the same developmental stage. In Brachypodium, as well as in other grasses such as wheat and rice, transitory starch accumulated in outer layers prior to endosperm cellularization until the beginning of grain development and then decline while the endosperm develops [3,4,59]. As a result of MLG loss, the enzymatic machinery for starch biosynthesis might be able to restart in the outer layers of the *cslf6*. Excepted for nucellar epidermis, MLG may play primarily a structural role in the outer layers to reinforce the hardness of grain. The shift toward starch in these layers is thus hardly explainable, especially since starch does not seem to be particularly accumulated in the epidermis of the nucellus. Investigation at later stages of grain development would be needed to deepen this issue.

## 4. Material and Methods

### 4.1. Plant Culture

The plants of *Brachypodium distachyon* accession Bd21.3 were grown in a culture chamber under a cycle of 16 h light at 24 °C and 8 h dark at 21 °C. Mature and dried grains were harvested for subsequent germination. For biochemical analysis of separated outer layers and endosperm, grains were harvested 15 days after flowering in order to be able to separate manually pericarp and endosperm which were then immediately frozen in liquid nitrogen prior analysis.

### 4.2. Seed Germination

To exclude the impact of lemma and palea on the germination rate, this structure was removed from the seeds prior to germination. Grains were soaked for 4 days in cold and sterile water for imbibition. Germination was conducted at 20 °C in 9 cm Petri dishes containing two layers of filter paper with 5 mL of distilled water. Two replicate dishes, each containing 50 seeds, were utilized for each treatment. Germinated seeds were collected at different times of germination and immediately frozen in liquid nitrogen for further analysis. Biochemical analyses were performed from halves of grains on the germ side (without germ).

### 4.3. Carbohydrate Analysis

#### 4.3.1. Preparation of Alcohol-Insoluble Residue (AIR)

Alcohol-insoluble residues (AIR) from Brachypodium grains previously crushed in liquid nitrogen were prepared by repeated extractions with hot ethanol solutions as described in [5]. Ethanol-washed materials was air dried and used for further biochemical analysis. All measurements were performed in triplicate.

#### 4.3.2. Neutral Sugar Determination

Soluble and AIR samples were subjected to complete acid hydrolysis using sulfuric acid 2 N (H_2_SO_4_) at 100 °C for 2 h, in triplicate. Inositol was used as internal standard. Individual neutral sugars were quantified after their derivatization into alditol acetates and gas chromatography analysis according to [60]. Total sugars resulted in the sum of peak area.

### 4.4. Starch and MLG Determination

Starch glucose was quantified in triplicate by HPAEC (CarboPac PA1 column 4 × 250 mm, Thermo-Fisher, Waltham, MA, USA) after amylolysis according to AOAC procedures (starch, AOAC method 996.11) with modifications described in [5].

MLG glucose was quantified in triplicate by HPAEC (CarboPac PA1 column 4 × 250 mm, Thermo-Fisher) after degradation of 5 mg AIR sample with Lichenase (Megazyme, Wicklow, Ireland) diluted at 20 U final as follows: 10 U Lichenase for 8 h at 40 °C and then additional 10 U Lichenase overnight at 40 °C to generate MLG derived oligosaccharides. Maltopentaose was used as an internal standard. The major DP3 and DP4 oligosaccharides were taken into account to estimate the quantity of MLG.

### 4.5. HPSEC Analysis

The half side of imbibed or germinated grains (on the germ side, without germ) were used for overnight alcali extraction of hemicelluloses with 1 M NaOH. Supernatants were then neutralized with acetic acid, digested with 2 U amyloglucosidase (Megazyme) 2 h at 40 °C. The reaction was stopped at 100 °C for 10 min, the samples were centrifuged and digested 30 min at 40 °C with 1.5 U endo-Xylanase NP (Megazyme) centrifuged and filtered on a PVDF 0. With glass prefilter 45 µm membrane (Millipore, Burlington, MA, USA) before to be injected (50 µL) on a Shodex OH-Pak-804 HQ column equipped with a Shodex OH-Pak SB-G pre-column. Elution was carried out at 0.7 mL/min with 50 mM sodium nitrate at 20 °C. A second digestion of the samples were performed with 10 U Lichenase (Megazyme) before being re-injected on the column.

### 4.6. Microscopic Analysis

#### 4.6.1. Histological Staining

Grains at different germination stages were fixed overnight in 3% (*w*/*v*) paraformaldehyde in 0.1 M phosphate buffer, pH7.4 dehydrated in ethanol series, and embedded with LR-White resin as described by Chateigner-Boutin et al., 2014 [61].

Transverse semi-thin sections (1 μm) of grains were cut with an ultracut (UC7, Leica, Deer Park, TX, USA) and stained either with Toluidine Blue O (1% in 2.5% Na_2_CO_3_ for 2 min then washed in water) and/or iodine solution (diluted 5 times for 2 min). Stained sections were observed using a Multizoom Macroscope (AZ100 M, Nikon, Amsterdam, Netherlands) under bright-field conditions.

#### 4.6.2. Immunolabeling

For immunofluorescence labeling, the semi-thin sections (1 μm) were successively incubated at room temperature in a blocking solution (3% (*w*/*v*) bovine serum albumin (BSA), 0.1 M Na-phosphate buffer saline (PBS), pH 7.2) for 30 min and in solutions containing primary antibodies for 1 h. The dilutions of the antibodies in PBS containing 1% (*w*/*v*) BSA and 0.05% (*w*/*v*) Tween-20 were: 1:20 for the mouse monoclonal INRA AX specific to arabinoxylans (produced in our laboratory [62]) and 1:200 for the mouse monoclonal antibody MLG (http://www.biosupplies.com.au, Bundoora, Australia). Cellulose-specific labeling was performed using His-tagged CBM3a at 10 µg/mL (obtained from Dr J.P. Knox, Centre for Plant Science, School of Biochemistry and Molecular Biology, Leeds University, England). After washes, grain sections were incubated with Alexa 546-conjugated secondary antibodies (Molecular Probes, http://www.invitrogen.com, Eugene, OR, USA) or monoclonal anti-polyhistidine antibodies produced in mouse (H1029, Sigma) diluted in PBS (1:100 (*v*/*v*)) for 1 h. The semi-thin sections were examined with a microscope (LEICA DMRD) equipped with epifluorescence irradiation. A band-pass filter 515–560 nm was used as excitation filter and fluorescence was detected at >590 nm. Before labeling using AX antibodies or CBM3a, the grain sections were pre-treated with Lichenase to remove (1–3) (1–4)-β-glucan (MLG) as previously described [33].

### 4.7. Cloning, Expression and Purification of Recombinant Lichenases

The genes Bradi2g22222, Bradi2g22224, and Bradi2g22226, coding for potential lichenases grouped in the *GH17* CAZy family, were amplified from Bd21.3 mRNA using the primers forward and reverse encompassing EcoRI and XbaI restriction site respectively and listed in Supplementary Table S1. The genes, deprived of their peptide signal, were cloned into the pPICZαA that contains an α-factor signal sequence downstream and an N-terminal fused six-histidine-tag and inserted in *Pichia pastoris X33 strain*. *Pichia pastoris X33* harbouring the pPICZαA-Bradi2g22222/24/26 was pre-cultured overnight in Yeast extract-peptone-glycerol (YPG) medium containing 100 μg/mL of zeocine (Invitrogen, Eugene, OR, USA) under shaking before being incubated in BMGY (Buffered Glycerol complexe medium) until reaching a DO_600 nm_ at around 50. Cells were harvested by centrifugation and resuspended in BMMY (buffered methanol complex medium), with the addition of 0.5% methanol every day for 4 days of cultivation at 30 °C under shaking at 300 rpm.

The supernatant was loaded on a 1 mL HisTrap^™^ FF column (GE Healthcare, Waukesha, WI, USA) to purify the His-tag GH17 proteins. The recombinant proteins were eluted with 20 mM Tris-HCl buffer pH 8 containing 200 mM NaCl, 300 mM imidazole. The eluted fraction from the HisTrap column was subjected to a 12% *w*/*v* polyacrylamide gel electrophoresis under denaturing conditions (sodium dodecyl sulfate polyacrylamide gel electrophoresis (SDS-PAGE)), together with a PageRuler™ Unstained Protein Ladder (Fisher, Illkirch, France) and then transferred to a nitrocellulose membrane to be subjected to western blot analysis using anti-His antibody (Sigma, Darmstadt, Germany). The supernatants containing the purified GH17 were buffer exchanged with Sodium phosphate 50 mM pH 6 and concentrated fifty times using a VIVASPIN 20 concentrator (PES membrane 10 kDa, Sartorius, Gottingen, Germany).

### 4.8. Protein Extraction

Total proteins were extracted from grains (without germ) and germs harvested at different time of germination. Fifty grains previously germinated in Petri dishes or just imbibed were grounded using a mortar and pestle in liquid nitrogen. Grain powder was washed 3 times in of cold citrate phosphate 100 mM pH 6 buffer by vortexing and centrifuging at 10,000 RPM 10 min at 4 °C. Proteins were extracted with cold citrate phosphate NaCl 1 M pH7 for 3 days and supernatants were recovered by centrifugation at 10,000 RPM 10 min 4 °C prior to measure enzymatic activities.

### 4.9. Enzymatic Activities

One hundred thirty-five μL of polysaccharide solutions were incubated with fifteen μL of protein extracts from germinated grains and germs or purified recombinant GH17 (100 µg/mL). One hundred microliters of aliquot were withdrawn and the amount of reducing ends produced was quantified by Nelson method adapted to microplate [63] and read on a microplate reader at a DO of 600 nm. The amount of reducing ends was expressed in μmol/mL or U/mL using standard curve prepared with glucose. Controls were prepared similarly with appropriate buffers and polysaccharide solutions.

α-amylase activities were assayed by using Ceralpha kit (Megazyme) with modifications. Fifty μL of protein extracts from germinated grains were incubated with 50 µL of HR solution (containing pNPG7 substrate and α-glucosidase) for 10 min at 40 °C. The incubation was stopped by adding 0.1 mL of 1 M sodium carbonate solution in an Elisa plate and optical density was measured at 405 nm. Negative control was prepared by replacing protein extract by extracting buffer (citrate phosphate NaCl 1 M pH7).

### 4.10. Quantitative RT-PCR

The extraction of RNAs from germinated grains (germ-free grains) was performed using a Plant RNA Mini-preps kit (Biobasic, Markham, Canada). After DNAse treatment, samples were purified and concentrated with Monarch RNA Clean Up kit (New England Biolabs, Ipswich, MA, USA). Reverse transcription was carried out with 0.5 μg of total RNAs, oligo d(T)23, and the ProtoScript First Strand cDNA Synthesis Kit (New England Biolabs).

Quantitative PCR was performed on the CFX Connect Real-Time instrument (Biorad, France) using SsoAdvanced Universal SYBR Green Supermix (Biorad, Hercules, CA, USA). Three replicates were performed for each experiment. The expression of genes of interest was normalized with two endogenous controls protein phosphatase 2A (PP2A) and succinate deshydrogenase (Bd-SDH) [64,65]. The relative expression values were calculated using the ∆∆*C*q method (software Bio-Rad CFX Maestro 2.0 (5.0.021.0616)). The primers used for RT-qPCR are listed in Supplementary Table S1.

## 5. Conclusions

Our work provided several lines of strong evidence (degradation of MLG during germination and the regulation and activity of MLG catabolic enzymes) demonstrating that MLG serves as the primary source of glucose during germination of Brachypodium seeds. This is in contrast to most grasses where starch serves as the source of glucose. While the remarkably low starch to MLG ratio in Brachypodium grain is consistent with the role of MLG as the primary energy source during germination, the question of why MLG accumulates rather than starch in Brachypodium grain is unresolved. The relatively easy depolymerization of MLG, only two enzymes for complete hydrolysis, make it as efficient as starch for storing glucose. Thus, plants have the flexibility of using either molecule and varying the ratio may be advantageous in some situations. This flexibility is highlighted by our observation that while disruption of the *CSLF6* gene leads to the total loss of MLG, germination is still possibly due to a shift to starch as the primary storage molecule in seeds. Astonishingly, the levels of the enzyme activities responsible for starch and MLG degradation during the germination were reversed in response to substrate changes in *cslf6* mutant plants. Together with previous works, these results suggest a co-regulation of MLG and starch biosynthesis whose mechanisms and involved actors are not yet known. Understanding the regulatory mechanisms governing carbon partitioning and metabolism in Brachypodium grain could provide an interesting way to control the overall nutritional value of grains, MLG of cereals being particularly beneficial for human health as a dietary fiber.

## Figures and Tables

**Table 1:** Sugar composition of the alcohol-insoluble residues (AIR) of wild type grains (WT, accession Bd21-3) at different stages of germination (time counted after the imbibition step). Results were obtained by gas chromatography analysis while the specific determination of starch and MLG contents was assayed by HPAEC analysis. The results are expressed as a percentage of AIR dry matter.

	Germination Time			
	0 h	24 h	48 h	96 h
Rhamnose	0.3 +/− 0.0	0.2 +/− 0.1	0.2 +/− 0.0	0.2 +/− 0.1
Fucose	ns	ns	ns	ns
Mannose	0.5 +/− 0.1	0.5 +/− 0.1	0.4 +/− 0.2	0.5 +/− 0.1
Galactose	0.6 +/− 0.0	0.7 +/− 0.0	0.6 +/− 0.0	1.0 +/− 0.1
Arabinose	3.1 +/− 0.2	3.0 +/− 0.0	3.3 +/− 0.3	5.3 +/− 0.0
Xylose	8.8 +/− 0.3	6.0 +/− 0.3	7.5 +/− 0.5	12.9 +/− 0.7
Glucose (total)	48.9 +/− 0.4	46.4 +/− 2.0	42.1 +/− 0.7	32.1 +/− 2.6
Starch	5.0 +/− 0.1	5.3 +/− 0.1	5.2 +/− 0.0	3.0 +/− 0.1
β-glucans	30.9 +/− 0.5	32.6 +/− 0.1	29.5 +/− 1.0	7.9 +/− 0.0
Total sugars	62.2 +/− 0.3	56.8 +/− 2.4	53.8 +/− 0.2	52.0 +/− 3.5

**Table 2:** Sugar composition of alcohol insoluble residues (AIR) of whole grains compared to separated endosperm and outer layers of *cslh1* and *cslf6* mutants and controls (WT and azygous *cslf6*) determined by gas chromatography analysis. Determination of starch and MLG content was carried out by HPAEC analysis. The results are expressed as a percentage of AIR dry matter. *cslf6*-ho: homozygous *cslf6* mutant; *cslf6*-az: azygous *cslf6* mutant.

	Mature Grain			Endosperm				Outer Layers			
	WT	*cslf6*-az	*cslf6*-ho	WT	*cslf6*-az	*cslf6*-ho	*cslh1*	WT	*cslf6*-az	*cslf6*-ho	*cslh1*
Rhamnose	0.3 +/− 0.1	0.3 +/− 0.0	0.2 +/− 0.0	0.4 +/− 0.1	0.3 +/− 0.1	0.3 +/− 0.0	0.3 +/− 0.0	0.3 +/− 0.1	0.5 +/− 0.1	0.2 +/− 0.0	0.4 +/− 0.0
Fucose	0.5 +/− 0.0	0.5 +/− 0.0	0.6 +/− 0.0	0.3 +/− 0.1	0.3 +/− 0.1	0.2 +/− 0.0	ns	0.3 +/− 0.0	0.4 +/− 0.0	0.3 +/− 0.1	ns
Mannose	ns	ns	ns	ns	ns	ns	ns	ns	ns	ns	ns
Galactose	0.6 +/− 0.1	0.7 +/− 0.2	1.6 +/− 0.1	1.2 +/− 0.1	0.7 +/− 0.3	1.5 +/− 0.3	0.8 +/− 0.2	1.0 +/− 0.2	0.8 +/− 0.2	1.9 +/− 0.1	0.6 +/− 0.0
Arabinose	2.9 +/− 0.3	3.0 +/− 0.1	7.3 +/− 0.1	2.2 +/− 0.2	2.2 +/− 0.2	3.5 +/− 0.1	2.5 +/− 0.0	4.3 +/− 0.1	4.3 +/− 0.4	8.5 +/− 0.3	4.1 +/− 0.0
Xylose	5.0 +/− 0.4	4.1 +/− 0.2	9.3 +/− 0.1	2.1 +/− 0.1	2.3 +/− 0.5	4.0 +/− 0.0	2.3 +/− 0.0	8.1 +/− 0.1	8.2 +/− 0.3	14.3 +/− 0.3	5.3 +/− 0.1
Glucose (total)	52.6 +/− 4.8	52.1 +/− 0.2	28.9 +/− 1.3	52.6 +/− 1.3	52.0 +/− 0.0	56.1 +/− 1.4	50.2 +/− 2.8	60.6 +/− 0.6	53.5 +/− 0.2	29.6 +/− 0.3	65.3 +/− 2.1
β-glucans	41.7 +/− 1.5	40.7 +/− 1.2	ns	46.8 +/− 0.1	48.5 +/− 1.1	ns	46.4 +/− 0.7	51.4 +/− 0.8	47.5 +/− 0.8	ns	50.0 +/− 0.2
Ratio BG3/BG4	5.4	5.1	ns	5.4	5.5	ns	4.9	4	4	ns	3.9
Starch	5.3 +/− 0.1	5.4 +/− 0.0	26.1 +/− 0.8	5.0 +/− 0.6	8.3 +/− 0.2	49.8 +/− 1.2	4.5 +/− 0.1	2.2 +/− 0.1	1.4 +/− 0.0	19.9 +/− 0.7	1.2 +/− 0.0
Total sugars	62.3 +/− 5.2	60.7 +/− 1.5	47.9 +/− 0.0	53.4 +/− 4.2	57.8 +/− 1.9	65.7 +/− 1.0	56.4 +/− 3.0	74.3 +/− 3.0	67.8 +/− 1.0	55.0 +/− 0.9	76.0 +/− 2.3

**Table 3:** Sugar composition of the alcohol-insoluble residues (AIR) of imbibed and germinated (96 h of germination) grains of the homozygous *cslf6* mutant determined by gas chromatography analysis. Determination of starch and MLG contents by HPAEC analysis. The results are expressed as a percentage of AIR dry matter. *cslf6*-ho: homozygous *cslf6* mutant.

	*csf6*-ho	
	0 h	96 h
Rhamnose	0.2 +/−0.0	0.2 +/−0.0
Mannose	0.7 +/−0.4	0.3 +/−0.2
Fucose	0.6 +/−0.0	0.6 +/−0.0
Galactose	1.5 +/−0.1	2.3 +/−0.1
Arabinose	6.4 +/−0.3	9.9 +/−0.1
Xylose	7.7 +/−0.3	18.0 +/−0.6
Glucose (total)	35.3 +/−5.0	21.5 +/−0.7
Starch	32.2 +/−0.2	7.2 +/−0.2
β-glucans	ns	ns
Total sugars	52.4 +/−4.8	52.9 +/−0.3

## Data Availability

Data is contained within the article or supplementary material.

## Supplementary Materials

The following supporting information can be downloaded at: https://www.mdpi.com/article/10.3390/ijms24076821/s1.

## References

[1] Wolny E., Betekhtin A., Rojek M., Braszewska-Zalewska A., Lusinska J., Hasterok R. (2018). Germination and the Early Stages of Seedling Development in *Brachypodium distachyon*. Int. J. Mol. Sci..

[2] Burton R.A., Fincher G.B. (2012). Current challenges in cell wall biology in the cereals and grasses. Front. Plant Sci..

[3] Guillon F., Larré C., Petipas F., Berger A., Moussawi J., Rogniaux H., Santoni A., Saulnier L., Jamme F., Miquel M. (2012). A comprehensive overview of grain development in *Brachypodium distachyon* variety Bd21. J. Exp. Bot..

[4] Trafford K., Haleux P., Henderson M., Parker M., Shirley N.J., Tucker M.R., Fincher G.B., Burton R.A. (2013). Grain development in *Brachypodium* and other grasses: Possible interactions between cell expansion, starch deposition, and cell-wall synthesis. J. Exp. Bot..

[5] Guillon F., Bouchet B., Jamme F., Robert P., Quemener B., Barron C., Larre C., Dumas P., Saulnier L. (2011). *Brachypodium distachyon* grain: Characterization of endosperm cell walls. J. Exp. Bot..

[6] Opanowicz M., Hands P., Betts D., Parker M.L., Toole G.A., Mills E.N.C., Doonan J.H., Drea S. (2011). Endosperm development in *Brachypodium distachyon*. J. Exp. Bot..

[7] Hands P., Drea S. (2012). A comparative view of grain development in *Brachypodium distachyon*. J. Cereal Sci..

[8] Fan M., Jensen J.K., Zemelis-Durfee S., Kim S., Chan J., Beaudry C.M., Brandizzi F., Wilkerson C.G. (2022). Disruption of *Brachypodium* Lichenase alters metabolism of mixed-linkage glucan and starch. Plant J..

[9] Woodward J.R., Fincher G.B. (1982). Substrate specificities and kinetic-properties of two (1→3),(1→4)-beta-D-glucan endo-hydrolases from germinating barley (*Hordeum vulgare*). Carbohydr. Res..

[10] Fincher G.B., Lock P.A., Morgan M.M., Lingelbach K., Wettenhall R.E.H., Mercer J.F.B., Brandt A., Thomsen K.K. (1986). Primary structure of the (1→3),(1→4)-beta-D-glucan 4-Glucanohydrolase from barley aleurone. Proc. Natl. Acad. Sci. USA.

[11] Litts J.C., Simmons C.R., Karrer E.E., Huang N., Rodriguez R.L. (1990). The isolation and characterization of a barley 1,3-1,4-Beta-Glucanase gene. Eur. J. Biochem..

[12] Slakeski N., Baulcombe D.C., Devos K.M., Ahluwalia B., Doan D.N.P., Fincher G.B. (1990). Structure and tissue-specific regulation of genes encoding Barley (1→3),(1→4)-beta-glucan endohydrolases. Mol. Gen. Genet..

[13] Slakeski N., Fincher G.B. (1992). Developmental regulation of (1-3,1-4)beta-glucanase gene-expression in barley—Tissue-specific expression of individual isoenzymes. Plant Physiol..

[14] Skendi A., Biliaderis C.G., Lazaridou A., Izydorczyk M.S. (2003). Structure and rheological properties of water soluble beta-glucans from oat cultivars of *Avena sativa* and *Avena bysantina*. J. Cereal Sci..

[15] Doblin M.S., Pettolino F.A., Wilson S.M., Campbell R., Burton R.A., Fincher G.B., Newbigin E., Bacic A. (2009). A barley *cellulose synthase-like CSLH* gene mediates (1,3;1,4)-beta-D-glucan synthesis in transgenic *Arabidopsis*. Proc. Natl. Acad. Sci. USA.

[16] Little A., Schwerdt J.G., Shirley N.J., Khor S.F., Neumann K., O’Donovan L.A., Lahnstein J., Collins H.M., Henderson M., Fincher G.B. (2018). Revised Phylogeny of the Cellulose Synthase Gene Superfamily: Insights into Cell Wall Evolution. Plant Physiol..

[17] Burton R.A., Jobling S.A., Harvey A.J., Shirley N.J., Mather D.E., Bacic A., Fincher G.B. (2008). The genetics and transcriptional profiles of the cellulose *synthase-like HvCslF* gene family in barley. Plant Physiol..

[18] Christensen U., Alonso-Simon A., Scheller H.V., Willats W.G.T., Harholt J. (2010). Characterization of the primary cell walls of seedlings of *Brachypodium distachyon*—A potential model plant for temperate grasses. Phytochemistry.

[19] Kim S.J., Zemelis S., Keegstra K., Brandizzi F. (2015). The cytoplasmic localization of the catalytic site of CSLF6 supports a channeling model for the biosynthesis of mixed-linkage glucan. Plant J..

[20] Ermawar R.A., Collins H.M., Byrt C.S., Henderson M., O’Donovan L.A., Shirley N.J., Schwerdt J.G., Lahnstein J., Fincher G.B., Burton R.A. (2015). Genetics and physiology of cell wall polysaccharides in the model C4 grass, *Setaria viridis* spp.. BMC Plant Biol..

[21] Burton R.A., Collins H.M., Kibble N.A.J., Smith J.A., Shirley N.J., Jobling S.A., Henderson M., Singh R.R., Pettolino F., Wilson S.M. (2011). Over-expression of specific *HvCslF* cellulose synthase-like genes in transgenic barley increases the levels of cell wall (1,3;1,4)-β-d-glucans and alters their fine structure. Plant Biotechnol. J..

[22] Kim S.J., Zemelis-Durfee S., Jensen J.K., Wilkerson C.G., Keegstra K., Brandizzi F. (2018). In the grass species *Brachypodium distachyon*, the production of mixed-linkage (1,3;1,4)-β-glucan (MLG) occurs in the Golgi apparatus. Plant J..

[23] Tonooka T., Aoki E., Yoshioka T., Taketa S. (2009). A novel mutant gene for (1-3,1-4)-beta-D-glucanless grain on barley (*Hordeum vulgare* L.) chromosome 7H. Breed. Sci..

[24] Cory A.T., Baga M., Anyia A., Rossnagel B.G., Chibbar R.N. (2012). Genetic markers for CslF6 gene associated with (1,3;1,4)-beta-glucan concentration in barley grain. J. Cereal Sci..

[25] Taketa S., Yuo T., Tonooka T., Tsumuraya Y., Inagaki Y., Haruyama N., Larroque O., Jobling S.A. (2012). Functional characterization of barley betaglucanless mutants demonstrates a unique role for CslF6 in (1,3;1,4)-beta-D-glucan biosynthesis. J. Exp. Bot..

[26] Hu G.S., Burton C., Hong Z.L., Jackson E. (2014). A mutation of the cellulose-synthase-like (*CslF6*) gene in barley (*Hordeum vulgare* L.) partially affects the beta-glucan content in grains. J. Cereal Sci..

[27] Wong S.C., Shirley N.J., Little A., Khoo K.H.P., Schwerdt J., Fincher G.B., Burton R.A., Mather D.E. (2015). Differential expression of the *HvCslF6* gene late in grain development may explain quantitative differences in (1,3;1,4)-beta-glucan concentration in barley. Mol. Breed..

[28] Nemeth C., Freeman J., Jones H.D., Sparks C., Pellny T.K., Wilkinson M.D., Dunwell J., Andersson A.A.M., Aman P., Guillon F. (2010). Down-regulation of the CSLF6 gene results in decreased (1,3;1,4)-beta-D-glucan in endosperm of wheat. Plant Physiol..

[29] Vega-Sánchez M.E., Verhertbruggen Y., Christensen U., Chen X., Sharma V., Varanasi P., Jobling S.A., Talbot M., White R.G., Joo M. (2012). Loss of Cellulose synthase-like F6 function affects mixed-linkage glucan deposition, cell wall mechanical properties, and defense responses in vegetative tissues of rice. Plant Physiol..

[30] Hsia M.M., O’Malley R., Cartwright A., Nieu R., Gordon S.P., Kelly S., Williams T.G., Wood D.F., Zhao Y., Bragg J. (2017). Sequencing and functional validation of the JGI *Brachypodium distachyon* T-DNA collection. Plant J..

[31] Bain M., van de Meene A., Costa R., Doblin M.S. (2021). Characterisation of Cellulose Synthase Like F6 (CslF6) Mutants Shows Altered Carbon Metabolism in β-D-(1,3;1,4)-Glucan Deficient Grain in *Brachypodium distachyon*. Front. Plant Sci..

[32] Vega-Sánchez M.E., Verhertbruggen Y., Scheller H.V., Ronald P.C. (2013). Abundance of mixed linkage glucan in mature tissues and secondary cell walls of grasses. Plant Signal. Behav..

[33] Francin-Allami M., Alvarado C., Daniel S., Geairon A., Saulnier L., Guillon F. (2019). Spatial and temporal distribution of cell wall polysaccharides during grain development of *Brachypodium distachyon*. Plant Sci..

[34] Granier F., Lemaire A., Wang Y., LeBris P., Antelme S., Vogel J., Laudencia-Chingcuanco D., Sibout R., Vogel J.P. (2016). Chemical and Radiation Mutagenesis: Induction and Detection by Whole Genome Sequencing BT. Genetics and Genomics of Brachypodium.

[35] Sibout R., Proost S., Hansen B.O., Vaid N., Giorgi F.M., Ho-Yue-Kuang S., Legee F., Cezart L., Bouchabke-Coussa O., Soulhat C. (2017). Expression atlas and comparative coexpression network analyses reveal important genes involved in the formation of lignified cell wall in *Brachypodium distachyon*. New Phytol..

[36] Dalmais M., Antelme S., Ho-Yue-Kuang S., Wang Y., Darracq O., d’Yvoire M.B., Cézard L., Légée F., Blondet E., Oria N. (2013). A TILLING Platform for Functional Genomics in *Brachypodium distachyon*. PLoS ONE.

[37] Tanackovic V., Svensson J.T., Jensen S.L., Buléon A., Blennow A. (2014). The deposition and characterization of starch in *Brachypodium distachyon*. J. Exp. Bot..

[38] Scholthof K.-B.G., Irigoyen S., Catalan P., Mandadi K.K. (2018). Brachypodium: A Monocot Grass Model Genus for Plant Biology. Plant Cell.

[39] Hands P., Kourmpetli S., Sharples D., Harris R.G., Drea S. (2012). Analysis of grain characters in temperate grasses reveals distinctive patterns of endosperm organization associated with grain shape. J. Exp. Bot..

[40] Selvig A., Aarnes H., Lie S. (1986). Cell-Wall Degradation in Endosperm of Barley During Germination. J. Inst. Brew..

[41] Langenaeken N.A., Ieven P., Hedlund E.G., Kyomugasho C., van de Walle D., Dewettinck K., Van Loey A.M., Roeffaers M.B.J., Courtin C.M. (2020). Arabinoxylan, β-glucan and pectin in barley and malt endosperm cell walls: A microstructure study using CLSM and cryo-SEM. Plant J..

[42] Oliveira L.A., de Souza G.A., Silva B.T., Rocha A.A.G., Picoli E.A.d.T., Pereira D.d.S., Donzeles S.M.L., Ribeiro M.d.F., Ferreira W.P.M. (2020). Histochemical approach of the mobilization of reserve compounds in germinating coffee seeds. Coffee Sci..

[43] Kosina R., Kaminska K. (2013). The role of nucellar epidermisduring the germination of *Brachypodium distachyon*. Annu. Wheat Newsl..

[44] Chateigner-Boutin A.L., Suliman M., Bouchet B., Alvarado C., Lollier V., Rogniaux H., Guillon F., Larré C. (2015). Endomembrane proteomics reveals putative enzymes involved in cell wall metabolism in wheat grain outer layers. J. Exp. Bot..

[45] Jerkovic A., Kriegel A.M., Bradner J.R., Atwell B.J., Roberts T.H., Willows R.D. (2010). Strategic distribution of protective proteins within bran layers of wheat protects the nutrient-rich endosperm. Plant Physiol..

[46] Buckeridge M.S., dos Santos H.P., Tine M.A.S. (2000). Mobilisation of storage cell wall polysaccharides in seeds. Plant Physiol. Biochem..

[47] Perrot T., Pauly M., Ramírez V. (2022). Emerging Roles of β-Glucanases in Plant Development and Adaptative Responses. Plants.

[48] Islam M.Z., An H.-G., Kang S.-J., Lee Y.-T. (2021). Physicochemical and bioactive properties of a high β-glucan barley variety “Betaone” affected by germination processing. Int. J. Biol. Macromol..

[49] Campbell J.M., Reid J.S.G. (1982). Galactomannan Formation and Guanosine 5’-Diphosphate-Mannose–Galactomannan Mannosyltransferase in Developing Seeds of Fenugreek (*Trigonella-Foenum-Graecum* L., *Leguminosae*). Planta.

[50] Mccleary B.V., Clark A.H., Dea I.C.M., Rees D.A. (1985). The Fine-Structures of Carob and Guar Galactomannans. Carbohydr. Res..

[51] Kumar C.S., Bhattacharya S. (2008). Tamarind seed: Properties, processing and utilization. Crit. Rev. Food Sci. Nutr..

[52] Dervilly G., Leclercq C., Zimmermann D., Roue C., Thibault J.F., Saulnier L. (2002). Isolation and characterization of high molar mass water-soluble arabinoxylans from barley and barley malt. Carbohydr. Polym..

[53] Akiyama T., Jin S., Yoshida M., Hoshino T., Opassiri R., Ketudat Cairns J.R. (2009). Expression of an endo-(1,3;1,4)-beta-glucanase in response to wounding, methyl jasmonate, abscisic acid and ethephon in rice seedlings. J. Plant Physiol..

[54] Lai D.M., Høj P.B., Fincher G.B. (1993). Purification and characterization of (1→3, 1→4)-beta-glucan endohydrolases from germinated wheat (*Triticum aestivum*). Plant Mol. Biol..

[55] Yun S.J., Martin D.J., Gengenbach B.G., Rines H.W., Somers D.A. (1993). Sequence of a (1-3,1-4)-beta-glucanase cDNA from oat. Plant Physiol..

[56] Kraemer F.J., Lunde C., Koch M., Kuhn B.M., Ruehl C., Brown P.J., Hoffmann P., Göhre V., Hake S., Pauly M. (2021). A mixed-linkage (1,3;1,4)-β-D-glucan specific hydrolase mediates dark-triggered degradation of this plant cell wall polysaccharide. Plant Physiol..

[57] Kim S.-J., Brandizzi F. (2021). Advances in Cell Wall Matrix Research with a Focus on Mixed-Linkage Glucan. Plant Cell Physiol..

[58] Purushotham P., Ho R., Yu L., Fincher G.B., Bulone V., Zimmer J. (2022). Mechanism of mixed-linkage glucan biosynthesis by barley cellulose synthase-like *CslF6* (1,3;1,4)-β-glucan synthase. Sci. Adv..

[59] Yu X.R., Zhou L., Zhang J., Yu H., Xiong F., Wang Z. (2015). Comparison of starch granule development and physicochemical properties of starches in wheat pericarp and endosperm. J. Sci. Food Agric..

[60] Dervilly G., Saulnier L., Roger P., Thibault J.-F. (2000). Isolation of homogeneous fractions from wheat water-soluble arabinoxylans. Influence of the structure on their macromolecular characteristics. J. Agric. Food Chem..

[61] Chateigner-Boutin A.-L., Bouchet B., Alvarado C., Bakan B., Guillon F. (2014). The wheat grain contains pectic domains exhibiting specific spatial and development-associated distribution. PLoS ONE.

[62] Guillon F., Tranquet O., Quillien L., Utille J.-P., Ordaz Ortiz J.J., Saulnier L. (2004). Generation of polyclonal and monoclonal antibodies against arabinoxylans and their use for immunocytochemical location of arabinoxylans in cell walls of endosperm of wheat. J. Cereal Sci..

[63] Sturgeon R.J., Dey P.M., Harb J.B. (1990). Methods in Plant Biochemistry.

[64] Molinari H.B.C., Pellny T.K., Freeman J., Shewry P.R., Mitchell R.A.C. (2013). Grass cell wall feruloylation: Distribution of bound ferulate and candidate gene expression in *Brachypodium distachyon*. Front. Plant Sci..

[65] Zhang K., Niu S., Di D., Shi L., Liu D., Cao X., Miao H., Wang X., Han C., Yu J. (2013). Selection of reference genes for gene expression studies in virus-infected monocots using quantitative real-time PCR. J. Biotechnol..

