# Supplementary material for: Mixed-Linkage Glucan Is the Main Carbohydrate Source and Starch Is an Alternative Source during Brachypodium Grain Germination
Source: Int J Mol Sci. 2023 Apr 6;24(7):6821. doi: 10.3390/ijms24076821 (PMC10095428; doi:10.3390/ijms24076821)
Supplement: Supplementary file 1 [file ijms-24-06821-s001.zip › ijms-2279056-supplementary.pdf]

Supplementary Figure S1

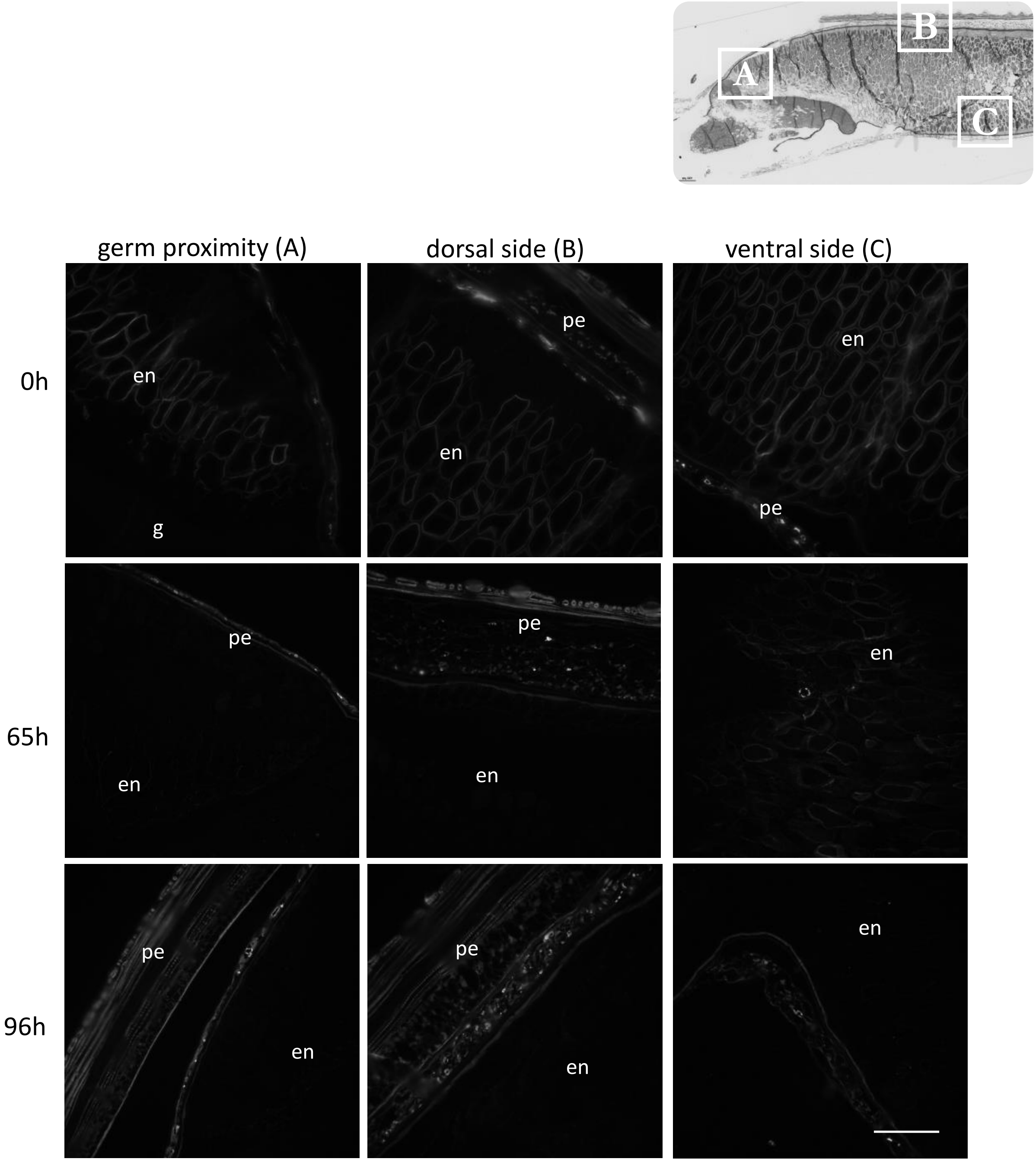

Immunolabeling control of wild type germinated grains (0, 65 and 96 hours after imbibition) pretreated with lichenase to remove MLG and using antibodies specific to MLG. Scale bar = 50µm. g : germ; pe : pericarp; en : endosperm.

Supplementary Figure S2

	GH17					GH51	GH9	GH16	GH13	CSLF6	CSLH1
	Bradi2g22222	Brad12g22224	Bradi2g22226	Bradi2g60490	Bradi2g27140	Bradi4g43710	Bradi5g14580	Bradi1g27867	Bradi3g58010	Bradi3g16307	Bradi5g10130
Pre-anthesis ovary	0.0	0.0	0.0	0.2	3.0	38.0	50.0	0.4	0.2	316.0	0.3
Young grain (1-3 DAA)	0.0	0.0	0.0	4.0	29.0	77.0	64.0	0.8	0.5	397.0	0.2
Middle length grain (3-8 DAA)	0.0	0.0	0.0	0.2	9.0	75.0	76.0	2.0	3.0	819.0	0.1
Full length grain (8-15 DAA)	0.0	0.0	0.0	0.1	27.0	62.0	89.0	4.0	12.0	940.0	0.1
Mature grain (15-20 DAA)	0.8	2.0	0.0	3.0	149.0	981.0	46.0	1.0	1.0	536.0	1.0
Mature grain without embryo	0.1	0.6	0.1	6.0	84.0	1000.0	33.0	2.0	2.0	618.0	0.9
Germinating grain	1052.0	2847.0	1520.0	849.0	329.0	1161.0	219.0	56.0	2223.0	213.0	2.0
Young seedling	0.1	2.0	4.0	19.0	468.0	46.0	148.0	43.0	0.2	498.0	10.0

expression<40
40<expression<100
100<expression<400
400<expression<800
800<expression<1500
expression>1500

Expression level (average expression values) of genes putatively involved in sugar metabolism of Brachypodium grain according to the *Brachypodium distachyon* eFP Browser (http://bar.utoronto.ca; Sibout et al., 2017 [35])

Supplementary Table S1

Primer lists used for gene cloning and qRT-PCR

	Accession	Forward primers	Reverse primers
primers used for cloning	Bradi2g22222	CCGGAATTCATCGGCGTGTGCTACGGC	GCTCTAGAGCAAATTTGATGGGGTAGATGTG
	Bradi2g22224	CCGGAATTCATCGGGGTGTGCTACGGC	GCTCTAGAGCCCAGAAGCTGAGGGGGTAGAC
	Bradi2g22226	CCGGAATTCATCGGGGTGTGCTACGGC	GCTCTAGAGCCCAGAAGCTGATGGAGTAGAC
primers used for qPCR	Bradi2g22222	CCAAAAGGCGTGGAGTCCATC	AGGCTGGCAAGCATGTCGTT
	Bradi2g22224	CCAAAAGCGTGGAGTCCATCG	CGACGACGTTGATGCCACTGC
	Bradi2g22226	CCAAAAGCGTGGAATCCATCG	TAGCGTGGGCAGCACGTCGTT
	Bradi2g27140	CACAAAGCGTGGAGTCGATCG	ATGTTGGAGAGCACGTCGTT
	Bradi2g60490	CTGTTCCTACGAGCGTGCAATC	GGCGATGTACTTGATGTTGACC
	Bradi4g43710	GAT AAC GAT CGC ACG TGG AAC C	CCGAAGTTCACAATCTTCACTCT
	Bradi5g14580	ACGCCCAGAAATCCGGCAAG	CCCTCACATGGTCGTACTCGC
	Bradi1g27867	GGCTTTCTACCTGTCGAACAACG	CAGAGGTGGAAGCGCATCTC
	Bradi3g58010	ACAAGGTCATGCAGGGCTAC	AGGTGCTCGATCTCCTCCTT
	PP2A	AGCGCTGGTGGAATCTGAAA	TGGACACGATCTAAGCACCG
	SDH	TTCAACAGCATGGATGGCC	ATCTTCGGTTGCAGAGCTCCT

Supplementary Figure S3

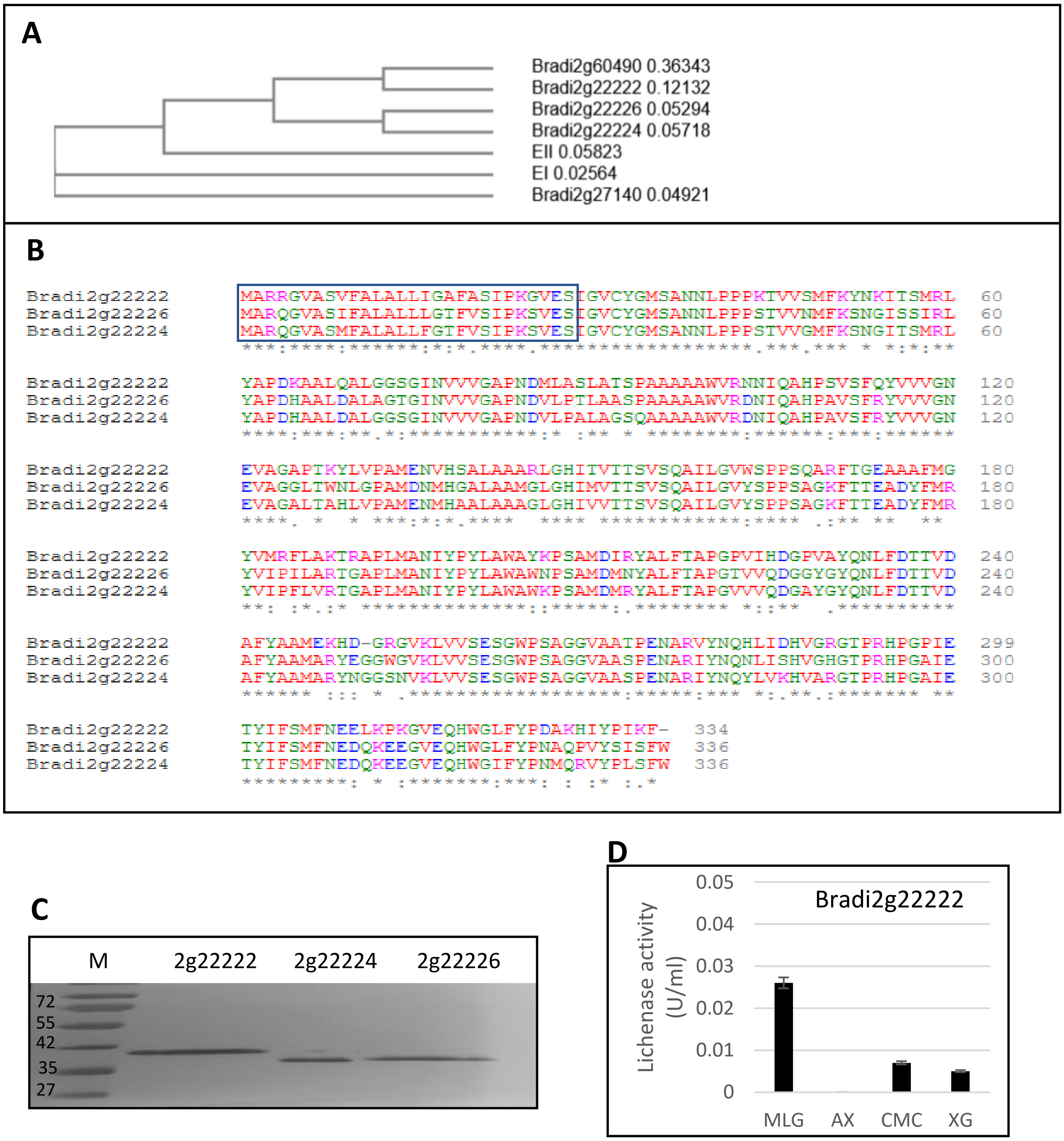

Lichenase information : phylogenetic tree, multiple alignment, western blot of recombinant lichenases, substrate specificity of Bd2g22222. A. Phylogenetic tree (https://www.ebi.ac.uk). EI : HORVU7Hr1G057680 ; EII : HORVU7Hr1G120450. **B**. Sequence alignment of three lichenases from the GH17 family using Clustal O (1.2.4) multiple sequence. Signal peptides is highlighted in a rectangular box. Amino acids are coloured according to their chemical properties (blue : acidic; red : small and hydrophobic ; magenta : basic ; green: hydroxyl, sulhydryl, amine and G). Asterisk significance : " *" indicates positions which have a single, fully conserved residue; ":" indicates conservation between groups of strongly similar properties; "." indicates conservation between groups of weakly similar properties. **C**. Western blot analysis of recombinant lichenases Bradi2g22222, Bradi2g22224 and Bradi2g22226 produced in *Pichia pastoris*. M : molecular weight marker (kDa). **D**. Enzymatic activity measurement of the recombinant enzyme Bradi2g22222 (MLG : barley mixed-linkage glucan; AX : wheat arabinoxylans; XG : tamarin xyloglucan, CMC : carboxymethylcellulose).

Supplementary Figure S4

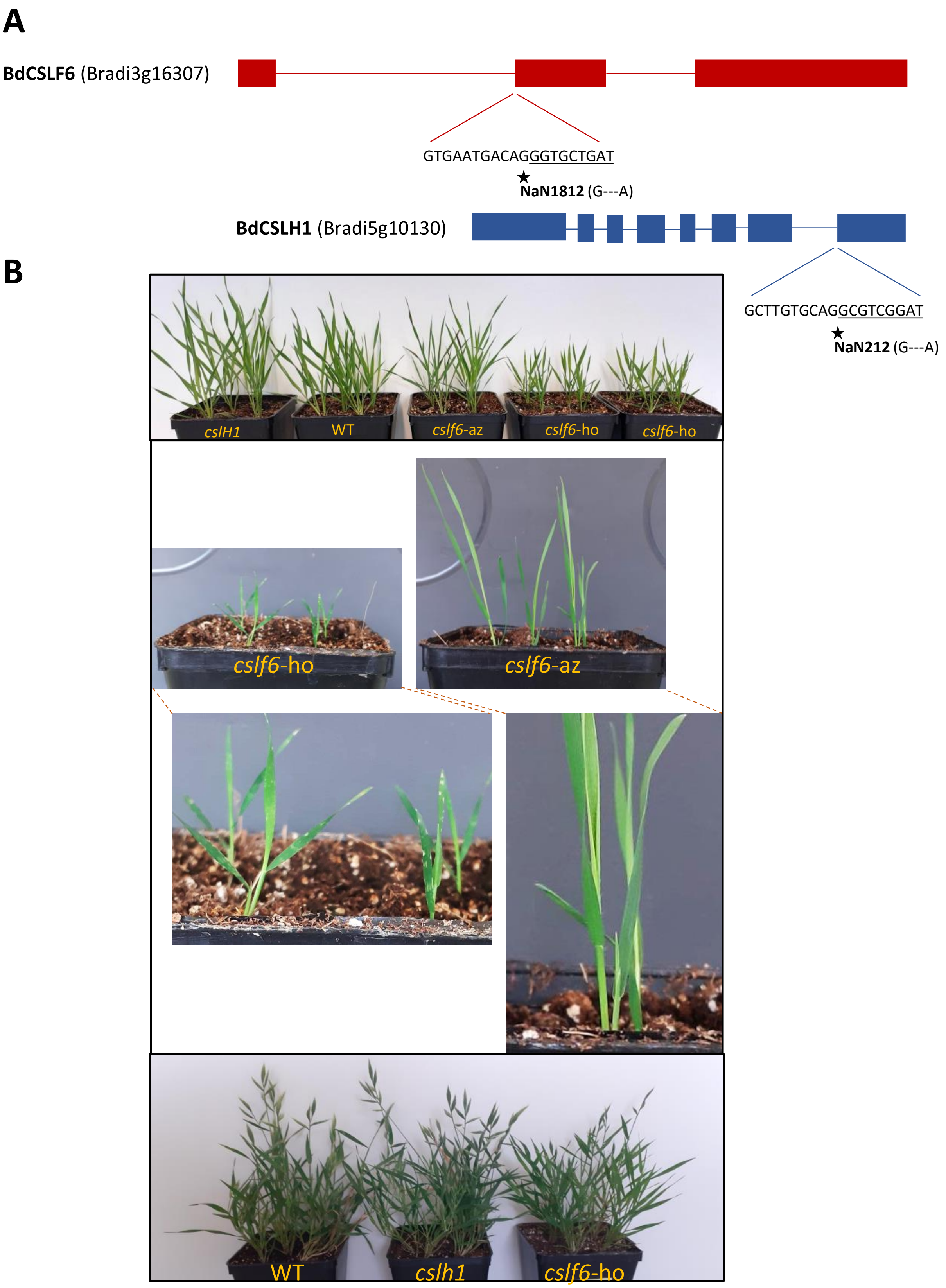

A. Schematic of Brachypodium *CSLF6* and *CSLH1* genes. Plain boxes correspond to exon sequences , and lanes correspond to intron sequences. Asterisk indicates the mutation location.
B. Brachypodium plants of WT, *cslh1* mutant, azygous and homozygous *cslf6* mutants showing young (top) and advanced growth (bottom), with magnifications (in the middle of the figure) highlighting leaf damage of the homozygous *cslf6* mutant.

Supplementary Figure S5

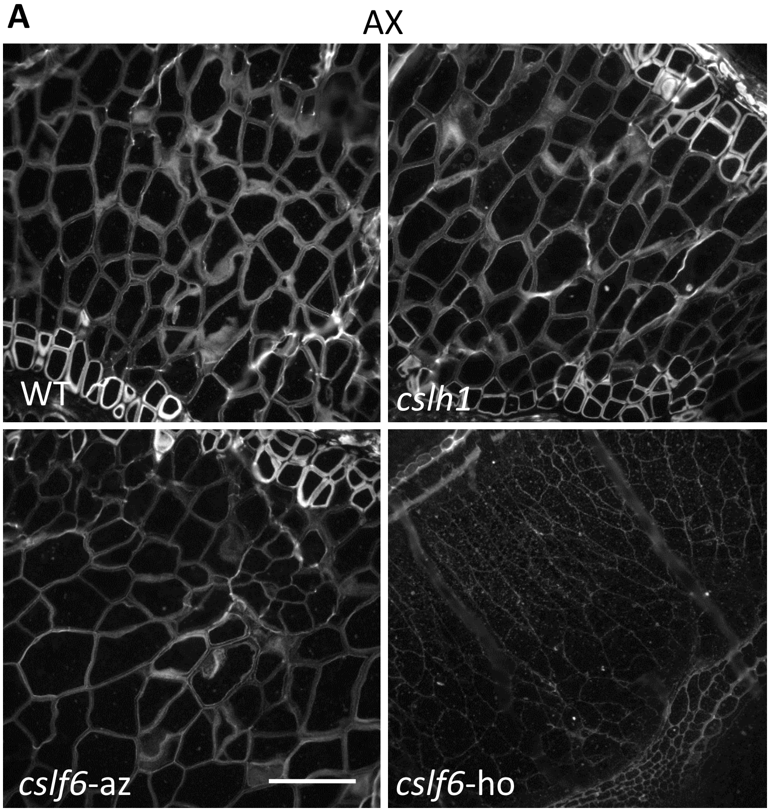

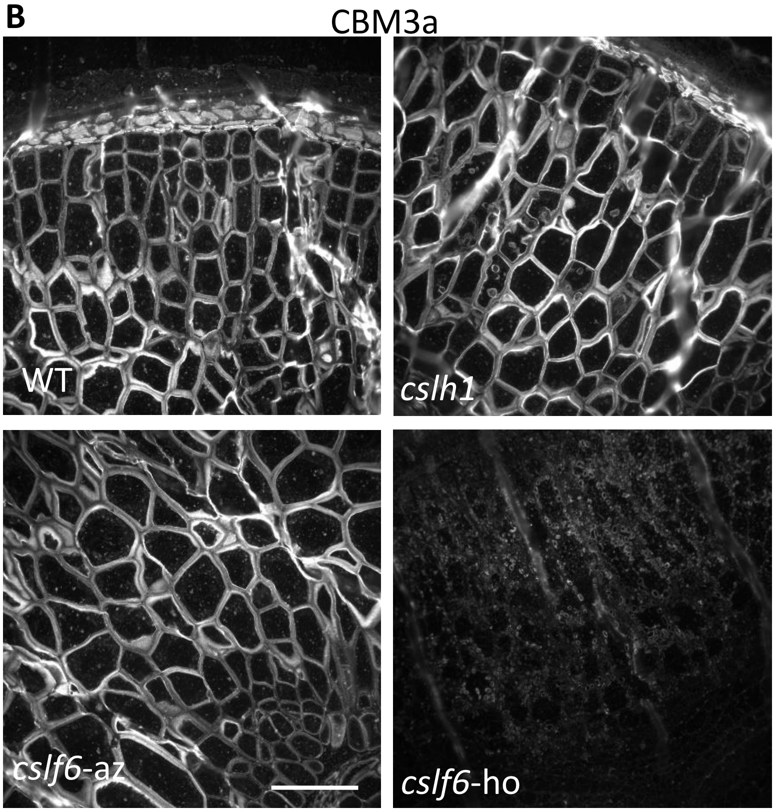

Fluorescence labeling of arabinoxylans and cellulose in grain cross-sections of WT, *cslh1*, homozygous and azygous *cslf6* mutants (15 days after flowering) pre-treated with lichenase, using AX1 antibodies (**A**) and CBM3a (**B**). Scale bar=50µm.

Supplementary Figure S6

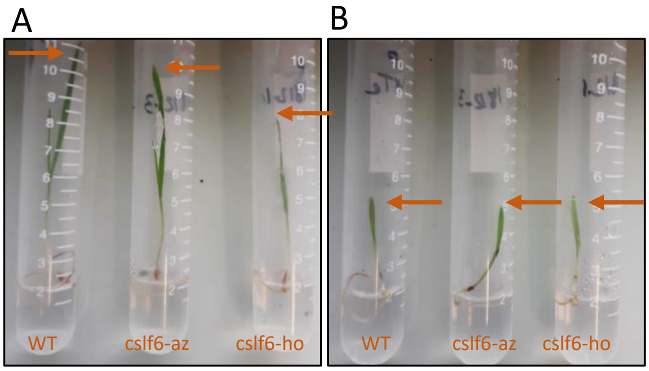

***In vitro* culture of WT and *cslf6* mutants**. Growth of plants on a phytagel medium from 8 days seedlings with seeds (**A**) or without seeds (**B**) on a phytagel medium (in light, without nutrient) during 6 days. n=15.
